# Role of soluble endoglin in BMP9 signaling

**DOI:** 10.1073/pnas.1816661116

**Published:** 2019-08-20

**Authors:** Aleksandra Lawera, Zhen Tong, Midory Thorikay, Rachael E. Redgrave, Jie Cai, Maarten van Dinther, Nicholas W. Morrell, Gijs B. Afink, D. Stephen Charnock-Jones, Helen M. Arthur, Peter ten Dijke, Wei Li

**Affiliations:** ^a^Department of Medicine, University of Cambridge, Cambridge CB2 0QQ, United Kingdom;; ^b^Department of Cell and Chemical Biology and Oncode Institute, Leiden University Medical Center, 2300 RC Leiden, The Netherlands;; ^c^Institute of Genetic Medicine, International Centre for Life, Newcastle University, Newcastle upon Tyne NE1 3BZ, United Kingdom;; ^d^Reproductive Biology Laboratory, Amsterdam University Medical Centers, University of Amsterdam, 1105 AZ Amsterdam, The Netherlands;; ^e^Department of Obstetrics and Gynaecology, University of Cambridge, Cambridge CB2 0SW, United Kingdom;; ^f^National Institute for Health Research, Cambridge Biomedical Research Centre, University of Cambridge, Cambridge CB2 0SW, United Kingdom;; ^g^Centre for Trophoblast Research, University of Cambridge, Cambridge CB2 3EG, United Kingdom

**Keywords:** BMP9, soluble endoglin, ALK1, placenta, preeclampsia

## Abstract

Endoglin (ENG) is a coreceptor of the transforming growth factor-β (TGFβ) family signaling complex, which is highly expressed on endothelial cells and plays a key role in angiogenesis. Its extracellular domain can be cleaved and released into the circulation as soluble ENG (sENG). High circulating levels of sENG contribute to the pathogenesis of preeclampsia (PE). Circulating bone morphogenetic protein 9 (BMP9), a vascular quiescence and endothelial-protective factor, binds sENG with high affinity, but how sENG participates in BMP9 signaling complexes is not fully resolved. sENG was thought to be a ligand trap for BMP9, preventing type II receptor binding and BMP9 signaling. Here we show that, despite cell-surface ENG being a dimer linked by disulfide bonds, sENG purified from human placenta and plasma from PE patients is primarily in a monomeric form. Incubating monomeric sENG with the circulating form of BMP9 (prodomain-bound form) in solution leads to the release of the prodomain and formation of a sENG:BMP9 complex. Furthermore, we demonstrate that binding of sENG to BMP9 does not inhibit BMP9 signaling. Indeed, the sENG:BMP9 complex signals with comparable potency and specificity to BMP9 on human primary endothelial cells. The full signaling activity of the sENG:BMP9 complex required transmembrane ENG. This study confirms that rather than being an inhibitory ligand trap, increased circulating sENG might preferentially direct BMP9 signaling via cell-surface ENG at the endothelium. This is important for understanding the role of sENG in the pathobiology of PE and other cardiovascular diseases.

## Significance

Endoglin (ENG) is a dimeric transmembrane accessory receptor highly expressed on endothelial cells. Its extracellular domain can be cleaved and released into circulation as soluble ENG (sENG). Higher levels of sENG contribute to the pathogenesis of preeclampsia (PE). Bone morphogenetic protein 9 (BMP9), a circulating signaling molecule and vascular quiescence factor, binds sENG with high affinity. Hence sENG has been thought to be a dimeric molecule and an inhibitory ligand trap for BMP9. Here we show that sENG purified from human tissues is monomeric and that the sENG:BMP9 complex is an active signaling molecule, but requires cell-surface ENG for optimal signaling. This study provides insight for understanding the role of sENG in PE and other cardiovascular diseases.

Endoglin (ENG) is a homodimeric transmembrane glycoprotein that is strongly expressed on endothelial cells (ECs) (1). Loss-of-function mutations in *ENG* cause hereditary hemorrhagic telangiectasia type I (HHT1) (2), which is characterized by telangiectases affecting the nose, gastrointestinal tract, and skin, as well as larger arteriovenous malformations (AVM) in the brain, lung, and liver. ENG mutations have also been reported in patients with pulmonary arterial hypertension (PAH), a vascular disorder characterized by the remodeling of small pulmonary vessels, resulting in increased right ventricular systolic pressure that ultimately leads to right-sided heart failure (3).

ENG has a large extracellular domain (ECD) and a short cytoplasmic tail, and its ECD can be cleaved from the cell surface under conditions related to endothelial dysfunction and inflammation (4). Cleaved ENG ECD, also known as soluble endoglin (sENG), is markedly elevated in preeclampsia (PE) and contributes to the pathogenesis of PE (5). Circulating sENG is also elevated in PAH and is proposed to be a biomarker for the prognosis of group I PAH patients (6). Intriguingly, administration of sENG reduces cardiac fibrosis in pressure overload-induced heart failure in mice (7).

In preclinical studies, loss of ENG leads to increased EC proliferation, decreased cell migration against flow, and reduced flow-mediated EC elongation (8–10). How ENG regulates such important cellular functions at the molecular level is not known. ENG was originally discovered as a component of the transforming growth factor-β (TGFβ) family signaling complex (11). TGFβ family ligands, including bone morphogenetic proteins (BMPs), are homodimers, initiating the cellular signaling by forming a signaling complex at the plasma membrane with 2 copies of a type I receptor and 2 copies of a type II receptor. TGFβ type I receptor (TGFβRI), also termed Activin receptor-like kinase (ALK)5, and TGFβ type II receptor (TGFβRII) mediate signaling from TGFβ1, -β2 and -β3, whereas ALK1 has been reported to participate in signaling in response to both TGFβ and BMPs (12, 13). ENG and betaglycan are coreceptors for the TGFβ family signaling, and both are single-pass transmembrane proteins (14). While their transmembrane and cytoplasmic domains show high sequence similarity (71% similarity with 63% identity in human), the extracellular domains of ENG and betaglycan share little sequence homology (11). While the coreceptor function of betaglycan is to capture and display TGFβ2 to its receptors (15), the molecular function of ENG is less well understood with many controversial reports and unanswered questions in the field. For example, using radio-labeled ligands and coimmunoprecipitation, ENG was initially identified as a component of the TGFβ receptor system, binding to TGFβ1 and TGFβ3 but not TGFβ2 (11); hence, sENG was proposed as a ligand trap for TGFβ1 (5). However, subsequent biochemical studies using purified recombinant ENG ECD-Fc fusion protein (ENG-Fc) revealed that ENG ECD binds directly with high affinity only to BMP9 and BMP10, but not to other TGFβ family receptors or ligands; hence, sENG has been proposed to be a ligand trap for BMP9 and BMP10 (16). Moreover, it has been shown that TGFβ1 can signal through ALK1 and ALK5 in endothelial cells, and its signaling through ALK1 requires ENG (12, 17, 18). However, ALK1 was later found to be a specific type I receptor for BMP9 and BMP10 (13, 19). Third, although ENG has been shown to inhibit TGFβ signaling (20), the requirement of ENG for BMP9 signaling has not been unequivocally established (21, 22).

BMP9 is synthesized by the liver and circulates at active concentrations in a prodomain-bound form (pro-BMP9) with its prodomain noncovalently bound to its growth factor domain (GFD) (23). The crystal structure of the sENG N-terminal orphan domain in complex with BMP9-GFD demonstrates that sENG binds to BMP9 at sites overlapping with the prodomain and the type II receptors (24–26). This implies that ENG ECD will need to displace the prodomain from BMP9 and then dissociate from BMP9 to allow type II receptor binding and the formation of the signaling complex. Using Biacore sandwich complex formation assays and measuring the changes in response units, it has been proposed that BMP9 prodomain can be displaced by the binding of ALK1, type II receptors, and sENG (27), although direct evidence from solution studies is yet to be shown. In order to assimilate the information into a coherent model of sENG function, a number of important questions remain unanswered, such as the physiological form of circulating sENG and whether sENG inhibits BMP9 signaling at physiologically relevant concentrations. The role of cell-surface ENG versus sENG function is also unclear.

To address these questions, we performed a detailed biochemical dissection of the protein–protein interactions between ENG ECD with BMP9 and its binding partners. We provide evidence that circulating sENG is primarily in the monomeric form and that monomeric sENG can readily displace the prodomain from pro-BMP9 to form a stable complex with BMP9. Moreover, sENG is not an inhibitory BMP9 ligand trap; instead, the purified sENG:BMP9 complex can signal in ECs with similar potency and specificity as pro-BMP9, but cell-surface ENG is required for optimal sENG:BMP9 signaling.

## Results

### Soluble Endoglin Purified from Human Placenta and Plasma Is Primarily Monomeric.

**Fig. 1.**
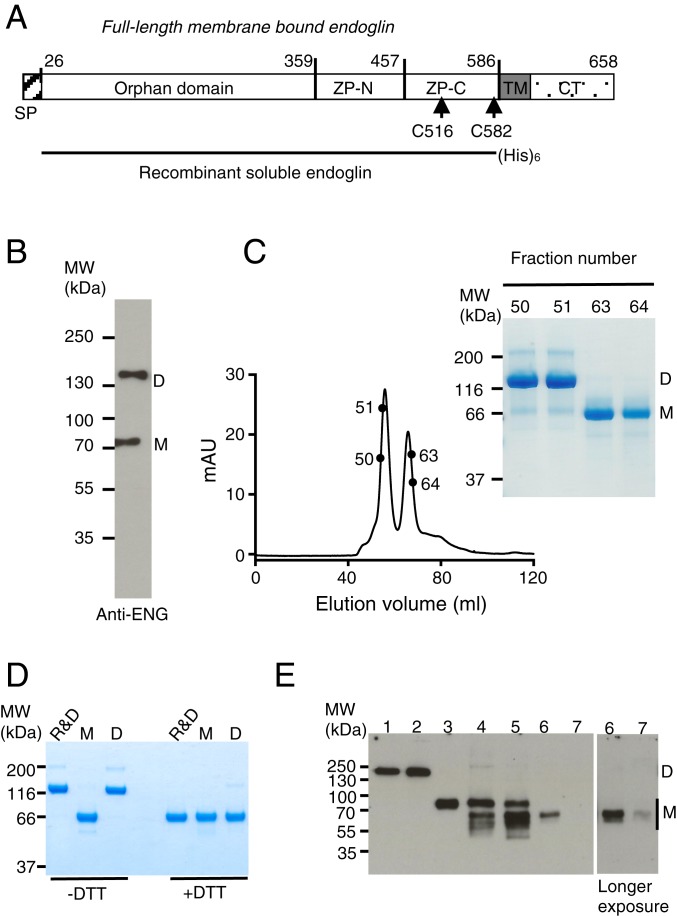
Generation and characterization of soluble endoglin. (*A*) Schematic illustrating the domain structure of human ENG and recombinant sENG. Cys516 and Cys582 participate in the intermolecular disulfide bond formation (24, 47). SP: signal peptide; TM: transmembrane domain; CT: cytoplasmic tail. (*B*) Soluble ENG expressed in the mammalian cells contains a mixture of dimer and monomer. Conditioned medium from HEK293-EBNA cells transfected with sENG construct was fractionated on a 4 to 12% Bis-Tris nonreducing SDS/PAGE and immunoblotted with anti-ENG antibody. (*C*) Fractions from the final S200 16/60 gel filtration column of sENG purification were run on a 12% nonreducing SDS/PAGE and stained with Coomassie Blue, showing the separation of sENG monomer (M) from dimer (D). (*D*) Comparison of in-house–purified sENG with that from R&D Systems by equal loading of purified proteins on an SDS/PAGE in the absence or presence of reducing reagent (DTT) and stained with Coomassie Blue. (*E*) Soluble ENG purified from biological sources is primarily monomeric. Soluble ENG from different sources was run on a 10% nonreducing SDS/PAGE and immunoblotted with anti-ENG antibody. Lane 1, sENG from R&D Systems; lanes 2 and 3, in-house–purified recombinant sENG dimer and monomer; lanes 4 and 5, sENG purified from conditioned media of 2 independently ex-vivo–cultured, human full-term fresh placenta from 2 healthy individuals; lanes 6 and 7, sENG purified from pooled plasma of PE (lane 6) and normotensive pregnant women (lane 7). Note that recombinant sENGs shown here are all with C-terminal His-tag. Similar results were obtained for a nontagged recombinant sENG which are shown in *SI Appendix*, Fig. S1. The apparent difference of the sENG sizes between Coomassie Blue-stained gel and Western blots was due to the difference in molecular weight markers (*SI Appendix*, Fig. S1*C*).

In order to characterize sENG, we expressed full-length ECD of human ENG containing amino acid residues 1 to 586 with a C-terminal His-tag in HEK293-EBNA cells (Fig. 1*A*). Conditioned medium from transfected cells contains both dimeric and monomeric sENG, which could be separated by gel filtration chromatography (Fig. 1 *B*–*D*). Similar results could be obtained using a construct without the His-tag (*SI Appendix*, Fig. S1 *A* and *B*), confirming that the C-terminal His-tag does not interfere with the dimerization. To determine whether sENG from natural sources is in a dimeric or monomeric form, we purified sENG from conditioned medium of ex vivo cultured full-term human placenta from healthy individuals using an anti-ENG antibody affinity column. Placenta-derived sENG is predominantly in the monomeric form, with a mixture of full-length ECD and smaller fragments (Fig. 1*E*). Similarly, sENG purified from plasma samples of PE and normotensive controls is also predominantly in the monomeric form and is slightly smaller than the full-length recombinant ECD (Fig. 1*E*). This is consistent with a previous report that sENG from PE plasma has truncations from the C terminus of the ECD (5). To exclude the possibility that the anti-ENG antibody column preferentially binds sENG monomers over dimers, we showed that both dimeric and monomeric sENG bound to anti-ENG columns equally well (*SI Appendix*, Fig. S2*A*), even in the presence of BMP9 (*SI Appendix*, Fig. S2*B*).

### Monomeric Form of sENG Is Stable under Oxidized Conditions as in PE Plasma.

**Fig. 2.**
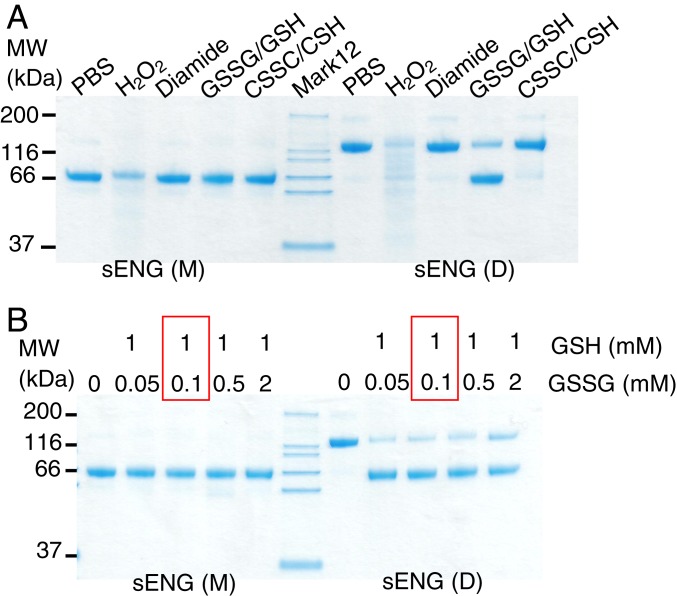
Monomeric sENG is stable under oxidizing environment. (*A*) sENG(M) is very stable under oxidizing conditions. Equal amounts of sENG monomer (M) or dimer (D) were incubated in either PBS or PBS with 1 mM hydrogen peroxide, 100 μM Diamide, 0.2 mM/2 mM GSSG/GSH, or 0.2 mM/2 mM cystine (CSSC)/cysteine (CSH) at 25 °C for 3 h. Samples were then subjected to 12% nonreducing SDS/PAGE and Coomassie Blue staining. The experiment was repeated 4 times and 1 representative gel is shown. (*B*) sENG(M) is very stable in GSH/GSSG redox buffer. Red box highlights the most oxidized buffer condition tested in Zhou et al. (28).

Since plasma from PE patients comprises a more oxidizing environment than plasma from healthy controls (28), we next investigated which form of sENG is more stable in such an environment. We first tested whether oxidizing reagents known to promote disulfide bond formation (28–31) could switch sENG(M) into sENG(D). Surprisingly, none of the oxidizing reagents promoted intermolecular disulfide bond formation in sENG(M), whereas sENG(D) was very sensitive to the presence of oxidized glutathione (GSSG)/reduced glutathione (GSH) redox buffer. H_2_O_2_ caused degradation in both sENG(M) and sENG(D), with greater effects on sENG(D) (Fig. 2*A*). We next incubated both forms of sENG in GSSG/GSH buffers, spanning conditions more oxidized than those shown by Zhou et al. (28) (Fig. 2*B*, red box). Redox buffer has typically been used in the refolding of extracellular proteins to promote disulfide reshuffling and formation (30). Again, sENG(M) was very stable in all conditions tested, whereas sENG(D) tended to convert into the monomer (Fig. 2*B* and *SI Appendix*, Fig. S1*D*), suggesting that the intermolecular disulfide bond in sENG(D) is labile.

These results led us to question whether there are free cysteines present in sENG(M) and sENG(D). We performed free thiol modification using 3 complementary methods: First, PEG5000 maleimide (mPEG5K), which modifies free cysteines resulting in the addition of 5 kDa of molecular weight for each modification and a band shift on SDS/PAGE (28); second, 5-Iodoacetamido-Fluorescein (5-IAF), a sulfhydryl-reactive derivative of fluorescein that labels accessible free cysteines (32, 33) and allows the detection of labeled protein by fluorescence (29); third, Ellman’s reagent 5,5′-dithiobis-(2-nitrobenzoic acid) (DTNB) (34), which reacts with the accessible free -SH group and allows quantification of free thiols by the highly chromogenic compound TNB. As shown in *SI Appendix*, Fig. S3, despite positive and negative controls showing the expected thiol modification results, no difference was observed in free cysteine labeling between sENG(M) and sENG(D) using all 3 methods. mPEG5K and 5-IAF did not detect any accessible free cysteines, whereas DTNB, the smallest molecule of the 3 labeling reagents, could detect one free cysteine in both dimeric and monomeric sENG.

### Both Dimeric and Monomeric sENG Form Complexes with BMP9, and sENG Interacts with ALK1 ECD via BMP9.

**Fig. 3.**
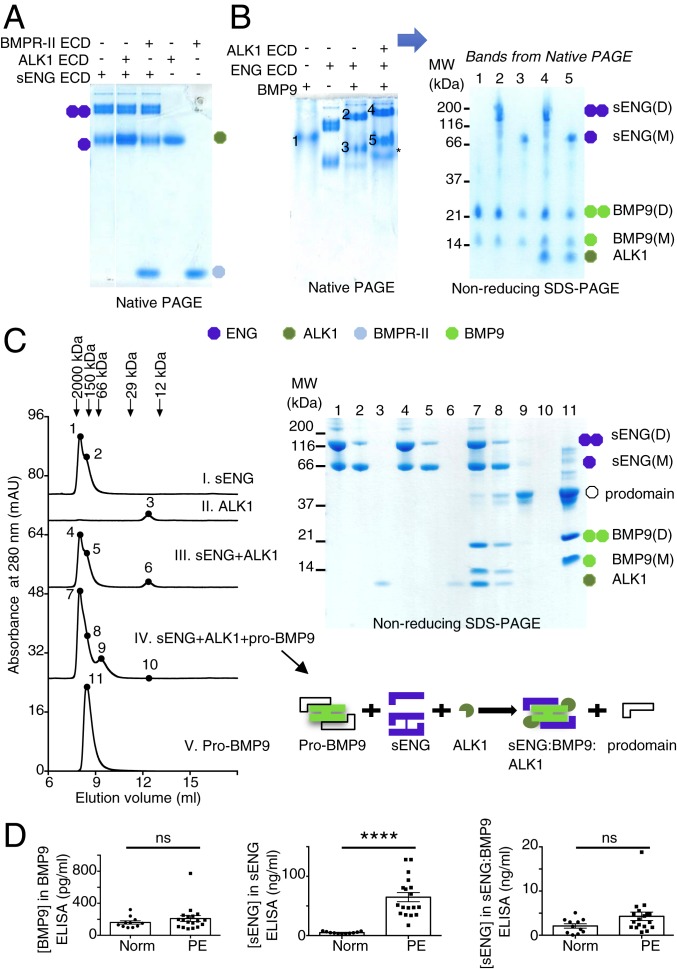
Soluble ENG binds BMP9 directly and interacts with ALK1 via BMP9. (*A*) No complex formation could be detected by native PAGE between sENG with either ALK1 or BMPRII ECDs. Samples in each lane were premixed as indicated and incubated at room temperature for 10 min before being loaded onto a 10% native PAGE. Two parts of the same gel are shown. The colored circles are used to label different proteins, with a single circle indicating a monomer and twin circles indicating the dimer. The key to the colored circles is shown below. (*B*) Complex formation analysis by native PAGE. (*Left*) Samples in each lane were premixed as indicated and incubated at room temperature for 10 min before being loaded onto a 10% native PAGE. Both dimer and monomer sENG can form complexes with BMP9, showing as band-shifts compared with sENG alone. The sENG:BMP9 complex can form a further complex with ALK1 ECD, showing as an additional band-shift. (*Right*) Bands 1 to 5 on the native PAGE were excised and rerun on a 12% nonreducing SDS/PAGE to confirm the protein components in each band. The band labeled with an asterisk (*) was not characterized in this experiment, but could be the complex between BMP9 and ALK1 ECD as observed previously (51). (*C*) sENG interacts with ALK1 ECD via BMP9. Purified sENG (a mixture of dimer and monomer), ALK1 ECD, and pro-BMP9 were run on a S75 10/30 gel filtration column, pre-equilibrated in buffer containing 50 mM Tris. HCl, pH 7.4, 150 mM NaCl, either by itself or in combination, as indicated. Eluted fractions from each run were collected and traces were overlaid (*Left*). Gel filtration standards were run, and the peak positions of each standard are shown above. Fractions were collected in each run, and at positions indicated as 1 to 11, fractions were precipitated by Trichloroacetic acid and run on a 12% nonreducing SDS/PAGE (*Right*). Identities of the proteins on the SDS/PAGE are labeled using colored circles as in *A*. A cartoon diagram, using the same color scheme as the circles, illustrates complex formation and the displacement of BMP9-prodomain. In the sENG:BMP9:ALK1 complex, sENG could be either monomer or dimer. (*D*) sENG:BMP9 complex can be detected in PE plasma. Plasma samples from 18 PE patients and 11 normotensive controls were subject to ELISA measurements for sENG, BMP9, and the sENG:BMP9 complex. Detailed methods and antibody pairs used in the ELISA measurements are described in *SI Appendix*, *Materials and Methods*. All measurements were carried out blind to the patient groups. For sENG:BMP9 measurements, one data point was excluded, and the complete dataset and the criteria for the exclusion are discussed in *SI Appendix*, Fig. S5. Means ± SEM are shown, and data were analyzed by 2-tailed unpaired *t*-test, ****P ≤ 0.0001; ns, not significant.

Since sENG binds BMP9-GFD with high affinity (16), we next investigated how sENG interacts with other components of BMP9 signaling complexes. Using coexpression followed by pull-down assays, Saito et al. have shown that ENG, BMP9, and ALK1 can form a ternary complex and that BMP9 mediates the interaction between ENG and ALK1 (24). We set out to investigate such interactions using purified recombinant proteins. Using native polyacrylamide gel electrophoresis (PAGE), we were unable to detect any direct interaction between sENG and ALK1 or BMPRII ECD (Fig. 3*A*). However, when sENG was premixed with BMP9 before native PAGE, a clear band shift was observed (Fig. 3*B*), and an additional band shift was detected when ALK1 ECD was included in the mixture (Fig. 3*B*), indicating the formation of sENG:BMP9 and sENG:BMP9:ALK1 complexes, respectively. The identities of sENG:BMP9 and sENG:BMP9:ALK1 complexes on the native PAGE were confirmed by subsequent SDS/PAGE analysis of these bands excised from the native gel (Fig. 3*B*). To further demonstrate that sENG interacts with ALK1 via BMP9, we carried out an analytical gel filtration experiment (Fig. 3*C*). In the absence of BMP9, neither dimeric nor monomeric sENG coeluted with ALK1 ECD, whereas, in the presence of BMP9, sENG, and ALK1, ECD coeluted with BMP9. It is interesting to note that, upon mixing pro-BMP9 with sENG and ALK1 ECD in solution, the prodomain was displaced (Fig. 3*C*, lane 9 on SDS/PAGE). The displacement of the prodomain was investigated further (below).

We next questioned whether the sENG:BMP9 complex is present in vivo and could be detected in plasma samples from PE patients and normotensive controls. Since sENG from plasma is monomeric, we investigated only sENG(M) in this study. First, we established an ELISA specific for the sENG(M):BMP9 complex (*SI Appendix*, Fig. S4). Control experiments showed that this ELISA specifically detects the sENG(M):BMP9 complex, not sENG(M) or pro-BMP9 on their own, whereas a BMP9 ELISA could detect BMP9 in both the sENG(M):BMP9 complex and pro-BMP9 equally well. Similarly, the sENG ELISA could detect free sENG(M) and sENG(M) in complex with BMP9 equally well (*SI Appendix*, Fig. S4). Plasma samples from PE patients and controls were assayed using all 3 ELISAs. The clinical characteristics of the controls and PE patients are summarized in *SI Appendix*, Table S1. There was no difference in overall circulating BMP9 levels between PE patients and normotensive control subjects. However, concentrations of sENG were significantly higher in plasma from PE patients, consistent with previous findings (5). The sENG(M):BMP9 complex was present in both groups and slightly higher in the PE group (Fig. 3*D* and *SI Appendix*, Fig. S5).

### Binding of sENG to Pro-BMP9 Displaces the Prodomain.

**Fig. 4.**
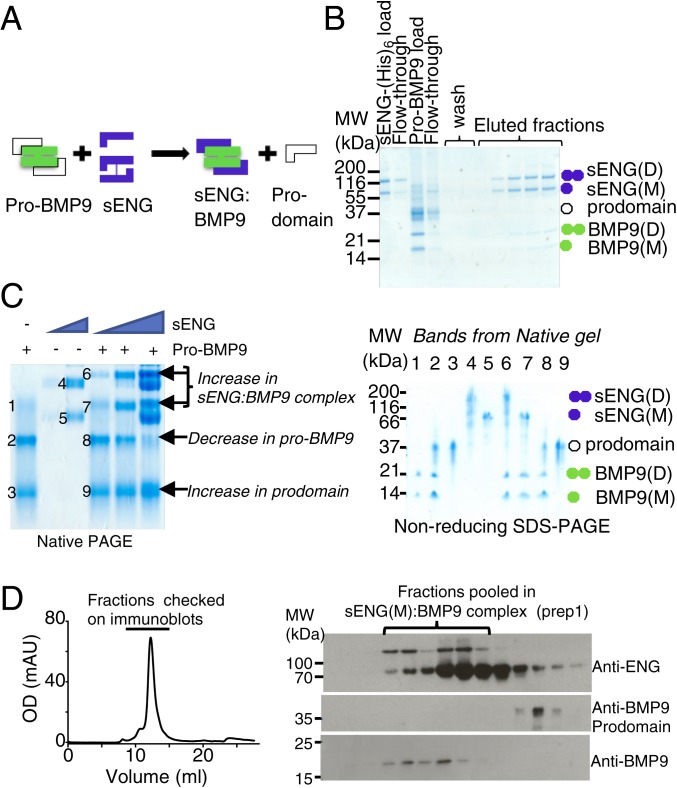
Binding of sENG to pro-BMP9 displaces the prodomain. (*A*) Cartoon illustrating the displacement of prodomain from pro-BMP9 complex upon sENG binding. Note that sENG dimer and monomer are not depicted in the sENG:BMP9 complex. (*B*) Prodomain displacement assay on a Ni-NTA column. Details are described in *SI Appendix*, *Materials and Methods*. Selected samples were run on a 12% SDS/PAGE and visualized by Coomassie Blue staining. Colored circles are used to indicate different proteins. (*C*) Complex formation analyzed by native PAGE. (*Left*) Native PAGE. (*Right*) Analysis of bands excised from native PAGE on a nonreducing SDS/PAGE. Colored circles are used to indicate different proteins. (*D*) Gel filtration analysis of prodomain displacement by monomeric sENG. Purified sENG(M) (1 mg) was mixed with pro-BMP9 (10 μg GFD) at room temperature for 30 min, and the mixture was then loaded onto an S200 10/30 gel filtration column pre-equilibrated in 20 mM Tris-Cl, pH 7.4, 150 mM NaCl. (*Left*) Gel filtration trace. (*Right*) Consecutive fractions run on a nonreducing SDS/PAGE and immunoblotted for the presence of sENG, BMP9 prodomain, or BMP9-GFD. Anti-BMP9 antibody preferentially reacts with BMP9 D-form on the nonreducing SDS/PAGE; hence, only one band is seen.

In order for circulating pro-BMP9 to bind ENG, the prodomain needs to be displaced (Fig. 4*A*). The importance of this interaction led us to extend our initial observation of prodomain displacement (Fig. 3*C*). We first performed a pull-down experiment using recombinant sENG (D+M) and pro-BMP9 (Fig. 4*B*). When pro-BMP9 was applied to a nickel-chelating column preloaded with His-tagged sENG, BMP9-GFD was retained on the column whereas the prodomain was in the flow-through (Fig. 4*B*). We also performed a native PAGE analysis with a follow-up SDS/PAGE confirming the identities of each band from the native PAGE. As shown in Fig. 4*C*, pro-BMP9 was separated as 3 bands on a native PAGE, corresponding to BMP9-GFD, the pro-BMP9 complex, and the prodomain alone. When pro-BMP9 was preincubated with an increasing amount of sENG before being loaded onto the native PAGE, the pro-BMP9 complex decreased with increasing amounts of sENG, with a concomitant increase in intensity of bands containing the sENG:BMP9 complex and prodomain, indicating further release of the prodomain from the pro-BMP9 complex by sENG (Fig. 4*C*). Finally, to confirm that the physiological form of sENG is capable of displacing the prodomain, we performed gel filtration with purified sENG(M) and pro-BMP9 and showed that sENG(M) could evict the prodomain in the absence of ALK1 (Fig. 4*D*). Altogether, these 4 independent solution-based studies provide compelling evidence that both sENG(D) and sENG(M) are able to readily and effectively displace the prodomain from BMP9.

### Soluble ENG Does Not Inhibit BMP9 Signaling at Physiological Concentrations.

**Fig. 5.**
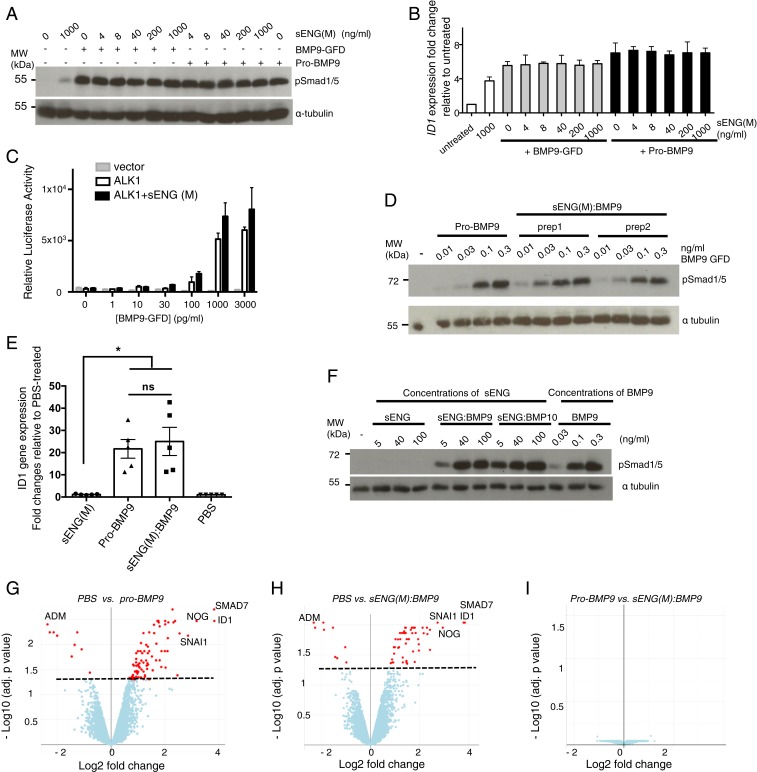
Soluble ENG does not inhibit BMP9 signaling at physiological concentrations. (*A* and *B*) sENG(M) does not inhibit BMP9 signaling in hPAECs. Serum-starved hPAECs were treated with BMP9-GFD or pro-BMP9, both of which had been preincubated with sENG(M) at indicated concentrations. Cells were harvested after either 15 min for pSmad1/5 immunoblotting analysis (*A*) or 1 h for RT-qPCR analysis (*B*). *n* = 3 for both immunoblot and qPCR analysis. Means ± SEM are shown in *B*. (*C*) sENG does not inhibit BMP9 signaling in C2C12 cells. Transfection of ALK1-sensitized C2C12 cells’ response to BMP9 at concentrations below 1,000 pg/mL. This ALK1-mediated BMP9 signaling, measured by a BMP-responsive element driven luciferase transcriptional reporter expression, was not inhibited by sENG(M) at 16 ng/mL. Means ± SEM are shown. (*D*) Purified sENG(M):BMP9 complex has similar potency to pro-BMP9 in inducing Smad1/5 phosphorylation. Serum-starved hPAECs were treated with pro-BMP9 alone or with 2 independent preparations of the sENG(M):BMP9 complex (*SI Appendix*, Fig. S7). After 15 min of treatment, cell lysates were harvested and subjected to immunoblotting analysis. Anti–α-tubulin was used as a loading control. (*E*) Purified sENG(M):BMP9 complex has similar activity to pro-BMP9 in inducing *ID1* gene expression. Five lines of hPAECs were serum-starved and treated with pro-BMP9 or sENG(M):BMP9 complex, all at 0.4 ng/mL BMP9-GFD concentrations, for 1.5 h. Cells were harvested and *ID1* gene expression was measured using RT-qPCR. Means ± SEM are shown, and data were analyzed by one-way ANOVA, Tukey’s posttest, *P < 0.05; ns, not significant. (*F*) At physiological concentrations of sENG, its complexes with BMP9 or BMP10 are active in inducing Smad1/5 phosphorylation. Serum-starved hPAECs were treated with sENG(M) alone, sENG(M):BMP9 complex (prep2), or sENG(M):BMP10 complex (*SI Appendix*, Fig. S7). After 15 min of treatment, cells were harvested and analyzed as above. All sENG concentrations were quantified using an SDS/PAGE and Coomassie Blue staining, using sENG from R&D Systems as standard. (*G*–*I*) sENG(M):BMP9 complex regulates the same set of target genes as pro-BMP9 in hPAECs. Serum-starved hPAECs were treated with PBS, pro-BMP9, or sENG(M):BMP9 at concentrations equivalent to 0.4 ng/mL BMP9-GFD. After 1.5 h, cells were harvested, and RNA was extracted for microarray analysis. Volcano plots showing the differential gene expression, with hits above the dotted line having the adjusted *P* value of less than 0.05.

Soluble ENG is often considered to be a ligand trap for BMP9 and BMP10 based on studies using ENG-Fc fusion protein (16, 35), but the effect of monomeric sENG (its natural circulating form) has not been investigated. We therefore performed the BMP9-mediated signaling assay in endothelial cells in the presence of sENG monomer from 4 to 1,000 ng/mL, which broadly spans the full range of concentrations measured in human plasma [3 to 4 ng/mL measured in healthy individuals to 40 to 150 ng/mL in PE patients (5, 6)]. In human pulmonary artery endothelial cells (PAECs), sENG(M), at all concentrations tested, did not inhibit Smad1/5 phosphorylation (Fig. 5*A*) nor *ID1* gene induction (Fig. 5*B*) induced by BMP9-GFD or pro-BMP9. To further determine whether sENG could act as a ligand trap and inhibit BMP9 signaling in another cell type, we performed BRE-luciferase reporter assays using C2C12 myoblast cells transfected with human ALK1. Smad1/5-dependent BRE-luciferase activity was potently induced by BMP9-GFD following ALK1 transfection, but this activity was not inhibited by sENG(M). This confirms that sENG is not an inhibitory ligand trap for BMP9 (Fig. 5*C*). Of note, using a similar concentration range, we did not find any evidence of sENG(D) inhibiting BMP9 signaling in PAECs (*SI Appendix*, Fig. S6 *A* and *B*). However, ENG-Fc, at a concentration of 1,000 ng/mL did partially inhibit pro-BMP9, but not BMP9-GFD–induced Smad1/5 phosphorylation (*SI Appendix*, Fig. S6*C*).

Since sENG can form a stable complex with BMP9 in solution, and sENG is not a ligand trap for BMP9, we went on to compare the signaling activity of the sENG(M):BMP9 complex with the circulating form of pro-BMP9. Two preparations of sENG(M):BMP9 complexes were generated using different protocols (*SI Appendix*, Fig. S7). BMP9-GFD concentrations in each protein preparation were quantified by BMP9 ELISA, and signaling assays were performed using molar equivalent concentrations of BMP9-GFD. No difference in signaling potency in human PAECs was found between sENG(M):BMP9 and pro-BMP9 (Fig. 5 *D* and *E*). Similar results were obtained for sENG(D):BMP9 (*SI Appendix*, Fig. S6*D*). We next asked whether sENG(M):BMP9 has signaling capacity at the range of (patho)physiological concentrations of sENG (5 to 100 ng/mL). As shown in Fig. 5*F*, sENG alone did not initiate Smad1/5 phosphorylation, whereas both sENG(M):BMP9 and sENG(M):BMP10 (generated in parallel with sENG:BMP9, *SI Appendix*, Fig. S7*B*) displayed potent signaling activity in the pathophysiological range of sENG concentrations.

In order to directly compare the downstream target genes regulated by the sENG(M):BMP9 complex and pro-BMP9, we profiled the hPAEC transcriptome induced by these 2 ligands using a microarray (36). For both treatments, we used the equivalent concentration of 0.4 ng/mL BMP9-GFD as a representative physiological concentration of BMP9 in healthy controls (37). The primary target genes were measured after 1.5 h of treatment. Comparison of pro-BMP9 or sENG(M):BMP9 with vehicle control (PBS) gave rise to a similar set of up- and down-regulated genes (Fig. 5 *G* and *H*), and no difference was detected when comparing genes regulated by pro-BMP9 versus sENG(M):BMP9 (Fig. 5*I*), again confirming that sENG did not interfere with BMP9 signaling and supporting the conclusion that sENG(M):BMP9 has similar signaling capacity to pro-BMP9 in endothelial cells. There were 94 transcripts significantly regulated by pro-BMP9 treatment and 61 by sENG(M):BMP9; these are listed in Datasets S1 and S2, respectively. There was almost complete overlap in the transcripts regulated by these 2 treatments (49, *P* = 1.3 × 10^−106^), and the magnitude of the transcript fold change elicited was highly correlated (*R*^2^ = 0.991, Dataset S3). This list of transcripts also overlaps with the 19 BMP9 target genes recently identified by Araki et al. (38).

### Coreceptor Function of the Cell-Surface ENG.

**Fig. 6.**
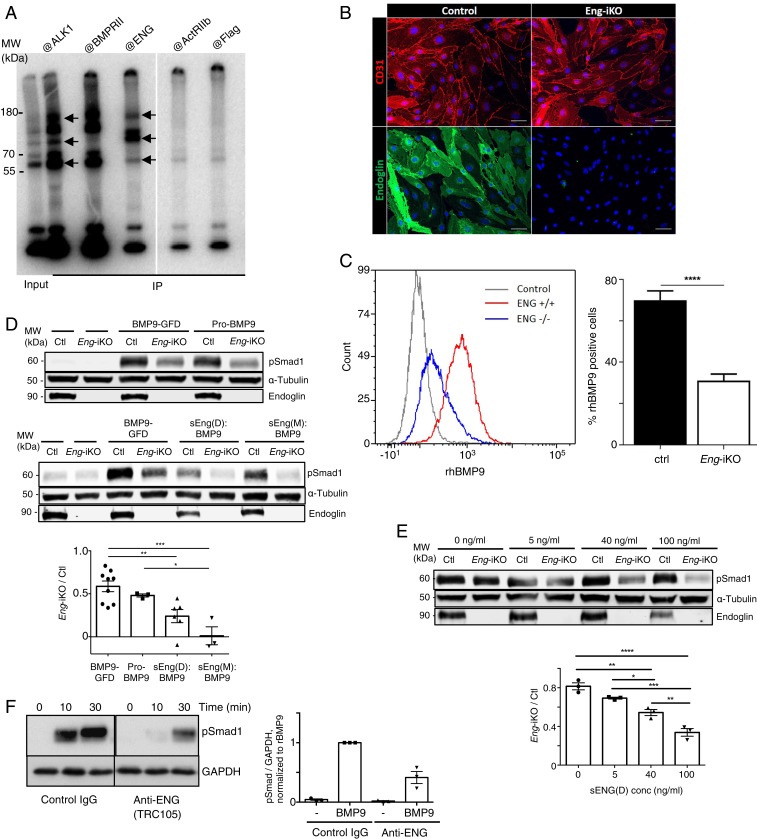
Coreceptor function of the cell surface ENG. (*A*) ALK1:BMP9:ENG complex can be detected on endothelial cell surface. Near confluent PAECs were incubated with ^125^I-BMP9 followed by chemical cross-linking and IP with antibodies indicated above the lanes. Two parts of the same gel are shown. Arrows mark the bands shared between ALK1 IP and ENG IP, indicating ternary complex formation. (*B* and *C*) ENG contributes significantly to the binding of BMP9 to the cell surface. (*B*) Confirmation of endoglin depletion in lung microvascular endothelial cells (MLECs) isolated from *Rosa26-Cre*^*ERT2*^*;Eng*^*fl/fl*^ mice. MLECs show positive CD31 staining with (control) and without (*Eng*-iKO) membrane ENG (Scale bar, 50 μm.) (*C*) Flow cytometry analysis shows that biotinylated recombinant human BMP9 (rhBMP9) binds with reduced efficiency to *Eng*-iKO endothelial cells compared with control. (*D*) *Eng-iKO* endothelial cells have reduced Smad1/5-dependent BMP9 signaling in response to recombinant BMP9 and pro-BMP9 compared with controls. This signaling is further reduced in *Eng-iKO* endothelial cells when BMP9 ligand is presented in complex with sENG(M) or sENG(D). (*E*) sENG inhibits serum-induced Smad1/5 phosphorylation more profoundly in *Eng-iKO* MLECs than in controls. For *D* and *E*, quantification of band intensities, means ± SEM are shown; one-way ANOVA, Tukey’s posttest was used for analysis. *P < 0.05; **P ≤ 0.01; ***P ≤ 0.001; ****P ≤ 0.0001. (*F*) Anti-ENG antibody TRC105 inhibits BMP9 signaling. TRC105 (10 μg/mL) or control IgG was preincubated with HMEC-1 cells for 5 h before BMP9-GFD was added to a final concentration of 0.1 ng/mL. After 10 or 30 min of treatment, cells were harvested for immunoblotting. Two parts of the same gel are shown. Densitometric quantification from the blots (30 min treatment, *n* = 3) is shown on the right.

In the final set of experiments, we asked whether the protein–protein interactions observed for sENG in solution could provide insights into the function of cell-surface ENG. First, we sought to confirm the presence of the ENG:BMP9:ALK1 complex on the endothelial cell surface. PAECs were cross-linked with ^125^I-BMP9, followed by immunoprecipitation (IP) using antibodies against several cell-surface receptors in turn. Common bands can be detected in anti-ALK1 and anti-ENG IPs which were not present in IP using the control antibodies anti-ActRIIb or anti-Flag (Fig. 6*A*), providing evidence for the presence of the ternary complex of ENG:BMP9:ALK1. Interestingly, strong bands were detected in anti–BMPRII-IP samples, suggesting that cell-surface BMPRII binds ^125^I-BMP9.

Next, we investigated the impact of cell-surface ENG expression on sENG function. Pulmonary endothelial cells from *Eng* floxed mice (Rosa26-Cre^ERT2^;Eng^fl/fl^) were prepared as in previous studies (39, 40), and *Eng* was depleted from endothelial cells in culture by transient treatment with 4-hydroxy-tamoxifen allowing the direct comparison of BMP9 signaling in control and *Eng* depleted (*Eng*-iKO) endothelial cells. Immunofluorescent staining confirmed that *Eng*-iKO endothelial cells were completely lacking ENG but still maintained endothelial CD31 expression indistinguishable from control cells (Fig. 6*B*). Incubating ECs with biotin-labeled BMP9-GFD followed by flow cytometric quantification of cell-surface bound biotin showed almost 50% reduction in BMP9 binding to cell surface in *Eng*-iKO ECs (Fig. 6*C*), consistent with a role for membrane ENG in providing a local supply of BMP9 ligand available to enhance signaling. In fact, *Eng*-iKO cells had almost 40% reduced pSmad1/5 signaling responses to both BMP9-GFD and pro-BMP9 (Fig. 6*D*). Interestingly, when sENG(M):BMP9 and sENG(D):BMP9 complexes were used as the source of BMP9 ligand, the response of *Eng*-iKO cells was even further depressed compared with control cells (Fig. 6*D*), suggesting that membrane ENG may be important in releasing BMP9 from the sENG:BMP9 complexes in order to initiate signaling. As serum is an important source of BMP9 in vivo, we also examined the effect of serum on signaling responses in *Eng*-iKO cells. In the presence of serum alone there is a small but consistent reduction in pSmad1/5/8 signaling in *Eng*-iKO compared with control ECs (Fig. 6*E*). With increasing concentrations of sENG(D) this effect escalates considerably such that pSMAD1/5 activation in response to serum in the presence of 100 ng/mL sENG(D) is reduced by ∼70% in the absence of membrane ENG, indicating that membrane endoglin is critical for making serum pro-BMP9 available for signaling in the presence of pathophysiological sENG concentrations. The role of cell-surface ENG in BMP9 signaling was further confirmed by the observation that an ENG antibody that blocks BMP9 binding to ENG (21) can inhibit BMP9-induced Smad1/5 phosphorylation (Fig. 6*F*).

## Discussion

In this report, we characterized recombinant sENG expressed from mammalian cells as well as that from ex vivo cultured placenta conditioned media and human normotensive and PE plasma samples. We found that, contrary to previous expectations, the circulating form of sENG is monomeric, even in PE patients, instead of the widely assumed S-S–linked dimer.

We also demonstrated here that, at physiological concentrations, sENG does not act as an inhibitory ligand trap for BMP9 and that the sENG(M):BMP9 complex can initiate Smad1/5-dependent signaling in human primary endothelial cells which is indistinguishable from that of the circulating form of pro-BMP9. Indeed, sENG treatment can reduce the incidence of AVMs in a mouse model of HHT1 (41), suggesting that it may enhance BMP9/10 signaling in vivo in the absence of endogenous ENG. However, we show here that the sENG:BMP9 complex requires membrane ENG for the most efficient signaling. It is also worth noting here that, in the setting of therapeutic intervention to target angiogenesis using ENG-Fc, which is an artificially dimeric form of sENG, it is possible for ENG-Fc to function as a ligand trap for BMP9 and BMP10.

We show here that monomeric sENG is very stable in a multiple oxidizing environment. From the crystal structures of ENG orphan domain and the ZP domain, only 4 cysteines, Cys242, Cys330 (not resolved in the structure), Cys516, and Cys582, in ENG ECD were not seen in the intramolecular disulfide bond form (24). It has been shown that a variant of ENG orphan domain lacking Cys242 and Cys330 is secreted as efficiently as wild type and retains BMP9-binding activity (24). Interestingly, a mammalian maltose-binding protein-fused ENG ZP domain, containing Cys516 and Cys582, was also expressed as a mixture of monomer and dimer, and crystallization selected the monomer form (24).

### Role of Monomeric sENG in the Onset of PE and Other Cardiovascular Diseases.

Our results shed light on 2 important questions regarding sENG: 1) How should results from studies using nonphysiological forms and concentrations of sENG be interpreted? 2) Is there a pathogenic role for sENG in PE and other cardiovascular diseases?

First, the in vivo form and concentrations of sENG need to be considered when investigating the role of sENG. Many studies have used high concentrations of sENG to study its pathophysiological effects, such as adenoviral-expressed sENG (5), transgenic overexpression of a truncated form of sENG (42–44), or ENG-Fc administration (5). Soluble ENG in such in vivo studies may not resemble the forms nor the concentrations found in humans and may not cause pathological effects through the same mechanism.

The pathogenic role of sENG in PE was initially thought to be because of its role as a ligand trap for TGFβ (5). Subsequent data showing the lack of direct binding of sENG to TGFβ have changed the view to sENG being the ligand trap for the high-affinity ligands BMP9 and BMP10 (16). Our data showing that sENG:BMP9 signals in hPAECs with similar potency to circulating pro-BMP9 refutes this. In addition, our data do not support the view that increased sENG alone is causal for endothelial dysfunction, which is in agreement with a recent report (45). Although the levels of sENG have been suggested as a biomarker of cardiovascular disease progression and treatment (6, 46), it is possible that changes in sENG levels are the consequences of the changes in the protein levels of cell-surface ENG which may also play a role in the disease pathogenesis. It has been shown that when sENG is elevated in circulation, increased cell-surface ENG is also detected (5, 7). Our data suggest that a small increase in circulating sENG concentrations in some patient groups, such as changes from ∼4 ng/mL in healthy controls to ∼8 ng/mL as measured in PAH patients (6), more likely reflects the consequence of endothelial dysfunction and increased shedding of cell-surface receptors, rather than sENG directly contributing to the disease pathogenesis.

### Coreceptor Function of ENG.

BMP9 and BMP10 are unique among the BMP family members in that they do not have the N-terminal heparin-binding region, and there is no known motif in BMP9 or BMP10 prodomains to suggest that they interact with an extracellular matrix. Therefore, one potential role of cell-surface ENG is to capture and present circulating BMP9 and BMP10, thereby increasing their local concentrations on the endothelial cell surface. This would be analogous to that of extracellular matrix and heparin for other BMPs (14), Indeed, we show that *Eng*-iKO endothelial cells have reduced capacity to bind BMP9 and compromised BMP9 signaling. The binding of sENG to form the sENG:BMP9 complex does not inhibit BMP9-initiated cellular signals, which could be due to either cells having intrinsic mechanisms to displace sENG to allow type II receptor binding and the formation of the canonical signaling complex or the type II receptors being able to directly form a complex with the ENG:BMP9 complex. The requirement of cell-surface ENG for mediating sENG:BMP9 signaling suggests that transmembrane ENG, but not type II receptors, can effectively displace sENG from the sENG:BMP9 complex.

**Fig. 7.**
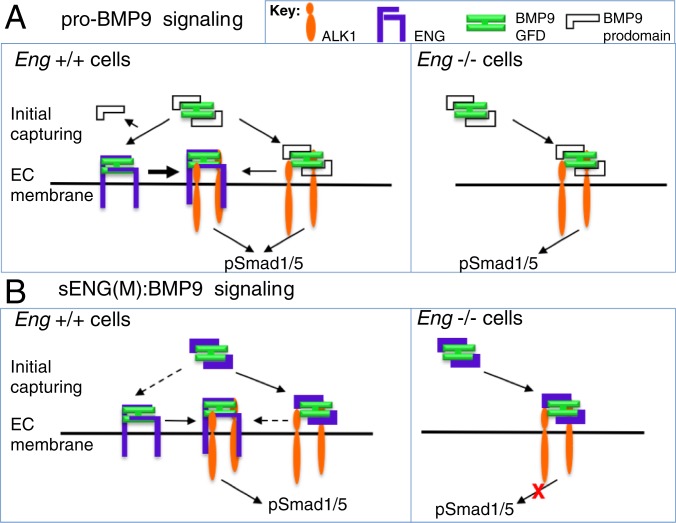
Model of sENG function in BMP9 signaling at the cell surface. (*A*) For pro-BMP9 signaling, high levels of ENG and ALK1 on endothelial cell surface capture the circulating pro-BMP9 effectively in *Eng* ^+/+^ cells, and the prodomain is displaced upon ENG binding. ENG and ALK1 can bind BMP9 simultaneously; therefore, the ternary complex of ENG:BMP9:ALK1 can be formed at the cell surface. In *Eng* ^−/−^ cells, pro-BMP9 can be captured only by cell-surface ALK1; therefore, less efficient capturing results in slightly reduced signaling. (*B*) sENG(M):BMP9 complex can initiate normal signaling in *Eng* ^+/+^ cells comparable to pro-BMP9, but not in *Eng* ^−/−^ cells, suggesting that cell-surface ENG is required for the signaling, presumably by displacing the sENG from the complex. Note that the stoichiometry of ENG and ALK1 is not depicted here, nor is the role of type II receptors.

Our data would support the following model for role of sENG in BMP9 signaling (Fig. 7). In the *Eng* ^+/+^ cells, circulating pro-BMP9 can be captured effectively by cell-surface ALK1 and ENG. While ALK1 can form a complex with pro-BMP9, the binding of ENG readily displaces the prodomain (Fig. 7*A*). When sENG is increased in circulation and forms an sENG:BMP9 complex (Fig. 7*B*), this complex can be captured by either cell-surface ENG or ALK1 in *Eng* ^+/+^ cells in a manner similar to that of pro-BMP9. Whereas in *Eng* ^−/−^ cells, although sENG:BMP9 can still be captured by cell-surface ALK1, the equilibrium is disrupted and Smad1/5 phosphorylation is reduced.

Since cell-surface ENG interacts with both BMP and TGFβ pathways (20, 47), it could potentially act as a stoichiometric link between the 2 pathways. The balance of endothelial BMP and TGFβ pathways has been shown to be essential in many cardiovascular diseases. For example, in PAH where BMP signaling is compromised in endothelial cells, TGFβ signaling is enhanced and inhibition of TGFβ signaling has been shown to offer therapeutic benefit in preclinical PAH models (48, 49). Interestingly, BMP9 increases endothelial cell ENG mRNA and protein expression (22), and ENG antagonizes TGFβ1 function in endothelial cells, indicating that ENG mediates the cross-talk between BMP and TGFβ signaling on endothelial cells. A more comprehensive knowledge of ENG and sENG interaction network is necessary before proper signaling assays can be designed to test these hypotheses. Furthermore, recent findings showing that sENG can also be found in the circulating microsomes (50) means that sENG may occur in additional membrane protein complexes, potentially adding further complexity to this network. Undoubtedly, because ENG is involved in many cardiovascular diseases and pathological angiogenesis, understanding the ENG interaction network and the ability to manipulate such protein–protein interactions will greatly facilitate the design of better therapeutic strategies targeting ENG in vascular diseases and cancer.

## Materials and Methods

### Human Samples.

The normal placental tissue collection was approved by the Cambridgeshire 2 Research Ethics Committee (reference number 07/H0308/163), and all participants provided written informed consent. Clinical data and blood samples were obtained from participants in the Preeclampsia and Non-Preeclampsia Database (PANDA) program of the Department of Obstetrics & Gynecology of the Academic Medical Center in Amsterdam, The Netherlands. Normotensive pregnant women and preeclamptic women with and without HELLP syndrome participating in the PANDA antenatal program were recruited consecutively. Experiments were carried out in accordance with the declaration of Helsinki after informed written consent from the participants was obtained and after experiments were approved by the ethics committee of the Academic Medical Center Hospital of the University of Amsterdam (AMC 2010_127).

Detailed materials and methods can be found in *SI Appendix*. Datasets reported here have been deposited in the GEO repository with the accession number GSE119206.

### Statistics.

All qPCR, transcriptional reporter, and Western blot experiments have been repeated at least 3 times, and representative experiments are shown. The statistical analyses were performed using a 2‐tailed, unpaired *t*‐test, or one-way ANOVA, as indicated in the figure legends. *P* < 0.05 was considered statistically significant.

## Supplementary Material

Supplementary File

Supplementary File

Supplementary File

Supplementary File
