# Supplementary material for: Role of soluble endoglin in BMP9 signaling
Source: Proc Natl Acad Sci U S A. 2019 Aug 20;116(36):17800–8. doi: 10.1073/pnas.1816661116 (PMC6731690; doi:10.1073/pnas.1816661116)

SI Appendix, Materials and Methods

Materials:

TRC105 (1, 2), a chimeric IgG1 monoclonal antibody that binds CD105/ENG, was a kind gift from TRACON and was used to make the affinity column and PAEC inhibition assay. Monoclonal antibodies against BMP9 (Cat No. MAB3209), biotinylated goat polyclonal anti-BMP9 antibody (BAF3209), and recombinant human sENG (Cat No. 1097-EN) and ENG-Fc (Cat No. 6578-EN) were purchased from R&D Systems. Antibodies against pSmad1/5 were purchased from Cell Signaling Technology or in-house raised polyclonal pSmad1/5 antibody (3). Anti-α-tubulin was from Sigma-Aldrich and anti-GAPDH from Millipore. Anti-human ENG antibody from BD Pharmingen (Cat No. 555690) was used in the immunoblots and ELISA. HiTrap NHS-activated HP column, HiTrap Excel column, HiTrap 5 ml Q HP column, protein A column, S200 16/60 gel filtration column, DMEM and CDCHO media were all purchased from ThermoFisher Scientific. CM Affi-Gel Blue Gel was purchased from BIO-RAD. Human pulmonary artery endothelial cells (hPAECs) and endothelial growth medium (EGM-2) were purchased from Lonza and PromoCell. Human embryonic kidney (HEK)293-EBNA were purchased from ATCC. All other chemicals were purchased from Sigma-Aldrich.

Generating recombinant His-tagged and non-tagged sENG

Human ENG cDNA, containing residues 1-586, was cloned between HindIII and BamHI sites in pCEP4 vector, with a C-terminal 6xHis tag. After confirming the sequence by DNA sequencing, the plasmid containing sENG was transiently-transfected into HEK293-EBNA cells in DMEM/5% FBS before changing into protein-free, serum free, chemically-defined CDCHO expression media. Conditioned media were harvested every 3-4 days and used for sENG purification.

To purify sENG, 5 litres of conditioned medium was loaded onto a 5 ml HiTrap Excel column pre-equilibrated in Buffer A (50 mM Tris-HCl, pH 7.4, 500 mM NaCl, 5 mM imidazole). After

extensive washing with buffer A, bound proteins were eluted using 5-1000 mM imidazole gradient in Buffer A. Fractions were run on a 12% SDS-PAGE and protein detected by Coomassie Blue staining, and those containing sENG were pooled and dialyzed against 50 mM Tris-HCl, pH 7.4, 50 mM NaCl before loading onto a 5 ml HiTrap Q HP column. After eluting the Q HP column with a NaCl gradient from 50 – 1000 mM, sENG-containing fractions were pooled and concentrated before loaded onto an S200 16/60 gel filtration column for separating sENG dimer and monomer.

For generating non-tagged sENG, a stop codon was introduced in the above construct after residue 586 and confirmed by DNA sequencing. The plasmid containing non-tagged sENG was transfected into HEK-EBNA cells and the conditioned medium harvested following the same protocol as above. The non-tagged sENG in the condition medium was captured using a HiTrap Q column. Fractions containing sENG, confirmed by western blot, were pooled and concentrated before fractionated on an S200 10/30 gel filtration column.

Purifying sENG from ex vivo cultured human placenta and plasma samples

Human placenta tissues from two donors were collected on the day of the delivery. Several small pieces of villi, about 2 cm in diameter, were isolated on ice, washed extensively in ice-cold sterile PBS before being cut into small pieces (~ 1 mm in diameter) and cultured in serum-free DMEM medium containing 1 mM vitamin C and 1 mM vitamin E in multiple T175 flasks. Conditioned medium was harvested every 24 hours up to 72 hours, cells debris removed by filtration and frozen at -20 °C immediately until purification.

TRC105 anti-ENG antibody column was generated by immobilizing TRC105 antibodies onto HiTrap NHS-activated HP column following manufacturer's instruction. *Ex vivo*-cultured placenta conditioned media was passed through the TRC105 column pre-equilibrated with PBS. After washes with 8 column volume of PBS, bound protein was eluted in fractions using 200 mM Glycine-HCl buffer, pH 2.2, and eluate neutralized immediately by collection into

tubes containing 1/8 volume of 1 M Tris-HCl, pH8.5. The presence of sENG in the fractions was confirmed by immunoblotting with anti-ENG antibodies.

To purify sENG from heparinized plasma, pooled plasma samples were initially passed through a 40 ml CM Affi-Gel Blue Gel column to remove albumin according to manufacturer's instruction, followed by protein A column to remove IgG, before being loaded onto the TRC105 column. Proteins bound to the TRC105 column were eluted and characterized in the same way as mentioned above.

Expression and purification of recombinant pro-BMP9

Human full-length proBMP9 cDNA was cloned into pCEP4 between XhoI and BamHI sites and verified by DNA sequencing. Plasmids containing proBMP9 were transfected into HEK293 EBNA cells using polyethylenimine as described previously (4). The purification method for pro-BMP9 was essentially the same as that described for pro-BMP10 (5).

BMP9 prodomain displacement assay on the nickel-nitrilotriacetic acid (Ni-NTA) column

His-tagged sENG, including a mixture of dimer and monomer, was loaded onto a Ni-NTA affinity column. After extensive washes, pro-BMP9 was loaded and flow-through fractions collected. After further washes, bound proteins were eluted with buffer containing 20 mM Tris-HCl pH7.4, 150 mM NaCl, 250 mM imidazole and fractions collected for gel analysis.

Generating monomeric receptor ECDs

The cloning of BMPRII ECD overexpression construct has been reported previously (5). The ALK1 ECD expression construct was generated by cloning a fragment of the human ALK1 gene (NM_000020) containing amino acids 22-118 into pET39b between NcoI and NotI sites. The expression and purification of ALK1 ECD followed the same protocol as previously described for BMPRII ECD (5).

Redox assays

To test the stability of sENG(M) and sENG(D) in the oxidizing conditions, purified His-tagged sENG monomer or dimer were incubated with PBS alone or PBS containing different oxidative regents, including 1 mM hydrogen peroxide, 100 μM diamide, 2 mM/0.2 mM reduced and oxidized form of glutathione (GSH/GSSG) or 2 mM/0.2 mM reduced and oxidized cysteine (CSH/CSSC), at 25 °C for 3 hours in the presence of protease inhibitor cocktail. Samples were run on a 12 % non-reducing SDS-PAGE gel and stained with Coomassie Blue.

For redox assay with different GSH/GSSG ratios, purified His-tagged or non-tagged sENG monomer/dimer were incubated with PBS alone or PBS containing 1 mM GSH with 0.05, 0.1, 0.5 and 2 mM GSSG at 25 °C for 24 hours in the presence of protease inhibitor (Complete-EDTA, Roche Diagnostics). Samples of His-tagged sENG were fractionated on 12% SDS-PAGE and stained with Coomassie Blue. Samples of non-tagged sENG were detected by western blot using anti-endoglin antibody. All gels were run under non-reducing conditions.

PEG5K maleimide (mPEG5K) labelling

Purified His-tagged sENG monomer and dimer, along with control proteins BSA, pro-BMP9 and pro-BMP10, were incubated with 1 mM mPEG5K (Sigma-Aldrich, Cat. No. 63187) or equal volume buffer (20 mM Tris pH6.5, 150 mM NaCl) at 25 °C for 2 hours in the presence of protease inhibitor (Complete-EDTA, Roche Diagnostics). After the reaction, samples were fractionated on a non-reducing 12% SDS-PAGE and visualized by Coomassie staining.

DTNB assay

DTNB (Ellman's Reagent, ThermoFisher, Cat. No. 22582) stock was prepared freshly in buffer A (0.1 M sodium phosphate, pH 8) at 20 mM. 25 μl of ENG (M) (stock conc. at 85 μM or 100 μM) and ENG (D) (stock conc. at 45 μM or 50 μM) along with positive and negative controls

were added to buffer A containing 400 μM DTNB to a final volume of 250 μl. All reactions were incubated for 20 min at room temperature in the dark. After incubation, 200 μl of each reaction was transferred into a 96-well flat bottom plate and measured at 415 nM in a plate reader. The following controls were used: BSA (Fisher Scientific, Cat. No. BPE9703), a linear peptide with two free cysteines (peptide 2 Cys): FFPCADDVTPTKHCIDQTLDHLK), and a linear peptide with no free cysteine (peptide 0 Cys): TKVGKASSVPTKLSPISVLYKDD). The concentration of free -SH group were calculated according to the standard curve of using L-Cysteine (Sigma-Aldrich, Cat. No. 168149) solutions. The ratio of free thiol to peptide monomer was calculated, and the number of free cysteine per peptide chain monomer was calculated by correcting the above ratio with the labelling efficiency of BSA.

5-Iodoacetamido-Fluorescein (5-IAF) labelling assay

5-IAF (Sigma-Aldrich, Cat No. I9271) was prepared as 100 mM stock in DMSO and protected from light. ENG(M) or ENG(D) (2 μg of each) was incubated with 5-IAF (at the same w/w ratio) in PBS (pH 7.4) for 2 hours at room temperature in the dark. Samples were run on a non-reducing 4-12% gradient SDS-PAGE (in the dark) and the gel was exposed under UV light to detect fluorescein labelling before subject to Coomassie staining. BSA, pro-BMP9 and pro-BMP10 were included as positive and negative controls.

Measuring BMP9, sENG and sENG:BMP9 complex by ELISA

Levels of sENG were measured in plasma using sENG ELISA kit from R&D system (Cat No. DNDG00). BMP9 ELISA was carried out using the monoclonal anti-BMP9 antibody (MAB3209) as the capture antibody and biotinylated goat polyclonal anti-BMP9 antibody (BAF3209) as the detecting antibody. Briefly, ELISA plates were coated with MAB3209 at 0.2 μg/well in PBS at 4°C overnight. After blocking with 1% BSA in PBS (BSA/PBS) for 2 hours at room temperature (RT), 25 μl plasma samples, diluted to 100 μl with BSA/PBS, were

added to each well and the plate incubated at room temperature for 2 hours at RT. After three washes with 0.05% Tween20 in PBS (PBS-T), BAF3209 (at 0.8 μg/well) was added and plate incubated at RT for 2 hours. After three washes with PBS-T, ExtrAvidin(r)-Alkaline phosphatase (Sigma) (diluted 1:400 in BSA/PBS-T) was added and incubated for 30 minutes at RT. Plates were washed with PBS-T followed by water and developed with a colorimetric substrate comprising 1 mg/ml 4-Nitrophenyl phosphate disodium salt hexahydrate (Sigma) in 1 M Diethanolamine, pH 9.8 containing 0.5 mM $MgCl_2$.

We developed an ELISA to measure the sENG:BMP9 complex as follows. To make the standard, sENG(M) and BMP9 were premixed at a molar ratio 10:1 in BSA/PBS overnight before being diluted to appropriate concentrations to make the standards, assuming all BMP9 is in the complex. Since the molecular weight of sENG is 5 times of that of BMP9, the values from this measurement were multiplied by 5 to obtain the sENG concentrations in the complex. Anti-ENG antibody (Bioscience) at 1 μg/ml was used to coat the ELISA plate overnight at 4°C. After washing and blocking with BSA/PBS, 100 μl of plasma sample or standard was added to each well and the plate incubated at RT for 2 hours. After washing with PBS-T, anti-BMP9 antibody (BAF3209 at 0.8 μg/ml) was added and plate was incubated for a further 2 hours before proceeding to the detection step as described for BMP9 ELISA.

Signaling assay in C2C12 cells

C2C12 mouse myoblast cells were grown in DMEM with 10% FBS and seeded in a 96-well plate. Transient transfection was carried out with a human ALK1 expression construct or empty vector using Lipofectamine (Invitrogen) according to the manufacturer's protocol. The cells were transfected with BRE-luciferase reporter construct (6) 24 hours later, and an expression plasmid for β-galactosidase was co-transfected and used to correct for transfection efficiency. Cells were serum-starved overnight before being stimulated for eight hours with the respective ligands the next day. Cells were washed, lysed and luciferase and β-galactosidase activity were

determined. Each transfection was carried out in triplicate and representative experiments are shown.

Signaling assays in endothelial cells

Smad1/5 phosphorylation in hPAECs and mouse lung microvascular endothelial cells (MLECs) were used to monitor the signaling activities of BMP9 and its complexes. After serum-starvation (hPAECs with overnight 0.1% serum or MLECs with 1 hour 0.5% serum), cells were treated with ligands at indicated concentrations as specified in the figure legends, before being harvested in SDS-containing lysis buffer. After quantification of total protein in the cell lysate, equal amounts of lysate were fractionated on a 12% SDS-PAGE and Smad1/5 phosphorylation detected by western blotting. Total α-tubulin or GAPDH was used as a loading control. Anti-endoglin (R&D System AF1320) was used to confirm endoglin depletion. Proteins were detected using either HRP and ECL for assays in hPAECs, or IR-Dye fluorescent secondary antibodies (Licor) and analysis using an Odyssey imaging system (Licor) for MLECs. Densitometric analysis was performed using ImageStudio software (Licor) or Image J.

Microarray experiment

Microarray analysis was carried out at Cambridge Genomic Services, the core facility at Department of Pathology, University of Cambridge. Four isolates of hPAECs were used (all from separate male subjects, Lonza). For each isolate, cells were grown to ~90% confluent in EGM-2, 10% FBS before serum-starvation overnight. Cells were then treated with BSA/PBS, sENG(M):BMP9 complex or pro-BMP9 alone for 1.5 hours and signaling was stopped by snap-freezing the cells in a dry-ice/ethanol bath. Total RNA was extracted using RNeasy Plus Mini Kit (QIAGEN). RNA quality was assessed using the bioanalyzer (Agilent Technologies, Cheadle, UK). The RNA integrity number (RIN) was 10 in each case. Reverse transcription

was carried out using High-Capacity cDNA Reverse Transcription Kit (Applied Biosystems). After quality control demonstrating the expected *ID1* gene induction in pro-BMP9 and sENG(M):BMP9 treated samples, the transcript profile was analyzed using the Affymetrix Gene ST 2.1 Array. Data were processed using the Robust Multichip Analysis (RMA) methodology (7), which carries out RMA background correction, quantile normalization and summarization. Once the data were processed, the comparisons were performed using the limma package (8) (Bioconductor) and the results corrected for multiple testing using False Discovery Rate (FDR) (9). Differentially expressed genes were visualized by plotting Significance (-log10(adjusted *P* value)) against logFC in a volcano plot. The microarray data have been deposited to GEO repository with the accession number GSE119206.

[^{125}I]BMP9 binding assay

Iodination of BMP9 was performed according to the chloramine T method and cells were subsequently affinity-labelled with the radioactive ligand as described before (10, 11). In brief, cells were pre-incubated with the radioactive BMP9 on ice for 3 hours. After incubation, cells were washed and crosslinking was performed using 0.27 μM disuccinimidyl suberate (DSS) and 0.7 μM bis (sulfosuccinimidyl) suberate (BS3, Pierce) for 15 minutes. Cells were washed and lysed. Cell lysates were incubated with the antibodies as indicated overnight and immune complexes were precipitated by adding protein A Sepharose (GE Healthcare). Samples were washed, boiled and subjected to SDS-PAGE. Gels were dried and scanned with the STORM imaging system (GE Healthcare).

Generating Eng-iKO endothelial cells

Mouse lines were maintained with approval from the local ethics committee. Primary mouse lung endothelial cells (MLECs) were isolated from lungs of *Eng$^{fl/fl}$;Rosa26-Cre^{ERT2}* mice carrying the Immortomouse transgene using CD31-coated dynabeads similar to previous work

(12, 13). Endothelial cells were cultured in endothelial MV2 media (PromoCell) containing 20 U/ml recombinant mouse interferon-γ (PeproTech) at 33°C. Addition of 2 μM 4-OH-tamoxifen (Sigma-Aldrich) for 48 hours (and hereafter in the absence of interferon) induced knock-out of the endoglin gene to generate *Eng*-iKO cells. All experiments were performed at 37°C at least 48 hours after tamoxifen was removed.

Flow cytometry of BMP9 binding to endothelial cells

Fluorokine Biotinylated Human BMP9 Kit (R&D Systems) was used as per manufacturer's instructions. Briefly, biotinylated recombinant human BMP9 was added to $1x10^6$ MLECs and incubated for 60 minutes at 4°C prior to addition of avidin-FITC for 30 minutes. Cells were washed and relative BMP9 binding capacity was then determined by flow cytometric analysis using an LSR II flow cytometer (BD).

SI Appendix, Table S1. Patient demographics and clinical parameters

Clinical parameter	Normotensive (n=11)	Preeclampsia (n=18)
Highest diastolic BP (mm Hg)	80 (70-92)	110 (100-125)
Urinary protein (g/24 h)	ND	6.0 (0.5-17.4)
HELLP	0%	58%
Gestational age at sample collection (d)	208 (175-269)	211 (174-258)
Gestational age at delivery (d)	246 (215-263)	212 (185-263)

Values are expressed as median (range) or percentage (%). ND=not determined.

References:

1. Nolan-Stevaux O, Zhong W, Culp S, Shaffer K, Hoover J, Wickramasinghe D, et al. Endoglin requirement for BMP9 signaling in endothelial cells reveals new mechanism of action for selective anti-endoglin antibodies. PLoS One. 2012;7(12):e50920.
2. Rosen LS, Hurwitz HI, Wong MK, Goldman J, Mendelson DS, Figg WD, et al. A phase I first-in-human study of TRC105 (Anti-Endoglin Antibody) in patients with advanced cancer. Clin Cancer Res. 2012;18(17):4820-9.
3. Persson U, Izumi H, Souchelnytskyi S, Itoh S, Grimsby S, Engstrom U, et al. The L45 loop in type I receptors for TGF-beta family members is a critical determinant in specifying Smad isoform activation. FEBS Lett. 1998;434(1-2):83-7.
4. Wei Z, Salmon RM, Upton PD, Morrell NW, Li W. Regulation of Bone Morphogenetic Protein 9 (BMP9) by Redox-dependent Proteolysis. J Biol Chem. 2014;289(45):31150-9.
5. Jiang H, Salmon RM, Upton PD, Wei Z, Lawera A, Davenport AP, et al. The Prodomain-bound Form of Bone Morphogenetic Protein 10 Is Biologically Active on Endothelial Cells. J Biol Chem. 2016;291(6):2954-66.
6. Korchynskyi O, ten Dijke P. Identification and functional characterization of distinct critically important bone morphogenetic protein-specific response elements in the Id1 promoter. J Biol Chem. 2002;277(7):4883-91.
7. Irizarry RA, Bolstad BM, Collin F, Cope LM, Hobbs B, Speed TP. Summaries of Affymetrix GeneChip probe level data. Nucleic Acids Res. 2003;31(4):e15.
8. Ritchie ME, Phipson B, Wu D, Hu Y, Law CW, Shi W, et al. limma powers differential expression analyses for RNA-sequencing and microarray studies. Nucleic Acids Res. 2015;43(7):e47.
9. Benjamini Y, Hochberg Y. Controlling the false discovery rate - a practical and powerful approach to multiple testing. Journal of the Royal Statistical Society Series B - Methodological. 1995;57(1):289-300.
10. Frolik CA, Wakefield LM, Smith DM, Sporn MB. Characterization of a membrane receptor for transforming growth factor-beta in normal rat kidney fibroblasts. J Biol Chem. 1984;259(17):10995-1000.
11. Yamashita H, ten Dijke P, Huylebroeck D, Sampath TK, Andries M, Smith JC, et al. Osteogenic protein-1 binds to activin type II receptors and induces certain activin-like effects. J Cell Biol. 1995;130(1):217-26.
12. Anderberg C, Cunha SI, Zhai Z, Cortez E, Pardali E, Johnson JR, et al. Deficiency for endoglin in tumor vasculature weakens the endothelial barrier to metastatic dissemination. J Exp Med. 2013;210(3):563-79.
13. Wang X, Abraham S, McKenzie JAG, Jeffs N, Swire M, Tripathi VB, et al. LRG1 promotes angiogenesis by modulating endothelial TGF-beta signalling. Nature. 2013;499(7458):306-11.

SI Appendix Figure Legends

Figure S1: Generation and characterization of non-tagged sENG. ***(A)*** Non-tagged sENG is secreted as a mixture of dimer and monomer. Conditioned medium from HEK293-EBNA cells transfected with His-tagged and non-tagged sENG constructs were fractionated on a 4-12% Bis-Tris non-reducing SDS-PAGE and immunoblotted with anti-His tag and anti-ENG antibodies. Note the anti-ENG blot of sENG-His is the same blot as shown in Figure 1B. ***(B)*** Non-tagged sENG dimer and monomer can be separated on a size exclusion column. Conditioned medium containing non-tagged sENG was captured by HiTrap Q column followed by gel filtration on a S200 10/30 column. Fractions 33-51 were run on a 7.5% SDS-PAGE under non-reducing conditions and blotted with anti-ENG antibody. ***(C)*** Unstained and pre-stained protein molecular weight markers used in this study, highlighting the difference in molecular weights of sENG observed on Coomassie stained gels and western blots was due to the protein markers used. ***(D)*** Non-tagged sENG monomer is stable in GSH/GSSG redox buffer. Samples were run on 4-12% Bis-Tris gradient gel under non-reducing condition.

Figure S2: Anti-ENG column controls. ***(A)*** Anti-ENG column binds to sENG dimer and monomer equally well. Purified sENG, containing a mixture of dimer and monomer, was loaded onto the anti-ENG column. After extensive wash with PBS, bound proteins were eluted in 0.1 M glycine buffer, pH 2.2. Samples from loaded material, flow-through, wash fractions and eluted fractions were run on a 12% SDS-PAGE and monitored by Coomassie Blue staining (top) or 10% SDS-PAGE for anti-ENG immunoblot (bottom). No difference in the dimer and monomer ratios could be observed in the loading and eluted fractions. ***(B)*** TRC105 column can bind to sENG dimer in the presence of BMP9. Purified sENG (125 μg), containing a mixture of monomer and dimer, was pre-incubated with 2x molar excess of pro-BMP9 at room temperature for 20 minutes before being loaded onto an anti-ENG column. After extensive washes with PBS, the column was eluted with 5 column volumes of 0.1 M glycine buffer at

pH 2.2. Samples from loaded material, flow-through, wash and eluted fractions were run on 10% SDS-PAGE and blotted for anti-ENG antibody, or 12% SDS-PAGE and blotted for anti-BMP9 monoclonal antibody.

Figure S3. Monomeric and dimeric sENG have similar accessibility for free thiol group under native conditions. (A) Positive and negative control proteins used in the thiol modification experiments: BSA with one surface exposed free cysteine, and BMP9 prodomain which has two cysteines forming a disulfide bond, therefore no free cysteine. (B) PEG5000 maleimide (mPEG5K) modification. Non-reducing 12% SDS-PAGE visualized by Coomassie staining. Positive control BSA could be modified with 1 free cysteine (new band marked with *) and negative controls BMP9 and BMP10 prodomains do not have any modification (**, no new band appearing after incubation with mPEG5K). Neither sENG(M) or sENG(D) could be modified by mPEG5K under such condition. (C) 5-iodoacetamido-fluorescein (5-IAF) modification assay. Non-reducing 4-12% SDS-PAGE visualized by Coomassie staining (left panel) or fluorescence (right panel) assesses the reactivity of free cysteine residues. Lanes with '+' indicate that material was incubated with 5-IAF just before electrophoresis. The labelled band is indicated with red *. Of note, control protein BSA in (B) and (C) runs at an apparent molecular weight smaller than expected. This is due to the non-reducing condition. BSA has 17 disulfide bonds and upon reduction, it runs at the expected molecular weight. (D) DTNB assay. Left: DTNB standard curve using L-cysteine. Right: number of free cysteines modified after correcting the labelling efficiency using BSA control. Peptides with 2 free cysteines (peptide 2 Cys) and 0 cysteines (peptide 0 cysteine) were used as positive and negative controls. Average from 4-5 measurements were plotted and means ± SEM are shown.

Figure S4. Controls ELISAs. ***(A)*** Generation of three test samples. Equal amounts of sENG(M) (50 ng/ml final concentration) and pro-BMP9 (10 ng/ml final GFD concentration)

were used to make three samples as listed and each sample was subjected to three control ELISA experiments for measuring sENG alone, BMP9 alone and sENG:BMP9 complex. In each ELISA, samples were applied at 2x, 4x and 8x dilutions and each dilution was measured in duplicate. ***(B)*** Soluble ENG ELISA. Note that the standard in the kit is the ENG dimer and hence when measuring ENG monomer, the measured level of 30 ng/ml is lower than the input which was quantified by SDS-PAGE and Coomassie Blue staining. Measurements from three dilutions were averaged and mean ± SEM is shown. ***(C)*** BMP9 ELISA. The mean value measured in three dilutions is 10 ng/ml, identical to the input. Measurements from three dilutions were averaged and mean ± SEM is shown. ***(D)*** ELISA specific for sENG(M):BMP9 complex. Since it was not known what percentage of sENG or BMP9 was in the complex form, it was not possible to generate the standard for sENG(M):BMP9 complex, hence direct OD values instead of calculated concentrations are shown. Dilution-dependent positive signals were detected for the sENG:BMP9 sample, whereas no signal above the background were detected for sENG or pro-BMP9 sample. Altogether, it can be seen that both sENG ELISA and BMP9 ELISA can detect the identical amount of sENG or BMP9 in the complexed and un-complexed samples respectively, suggesting that the formation of the sENG(M):BMP9 complex does not interfere with measurements of sENG or BMP9 by these ELISA.

Figure S5. Analysis of ELISA measurements from plasma samples. ***(A)*** Comparison of sENG levels measured by total sENG ELISA (red bars) and by sENG:BMP9 ELISA (white bars). ***(B)*** Comparison of BMP9 levels measured by total BMP9 ELISA (blue bars, left Y-axis) and by sENG:BMP9 ELISA (white bars, right Y-axis). The concentrations of BMP9 measured in BMP9 ELISA is about 2-10 fold lower than those measured in the sENG:BMP9 ELISA, and this was probably due to the binding limit of the current available BMP9 antibody pair since our control experiment using BMP9 spike into plasma samples showed 25-50% recovery of pro-BMP9 in such ELISA. The sENG concentrations obtained from the two ELISA

measurements gave results in a similar range, with sENG concentrations measured from sENG:BMP9 complex ELISA generally lower than the total sENG measured in sENG ELISA, in agreement with a fraction of sENG being in the complex with BMP9. One outlier can be seen in the sENG:BMP9 complex measurements (sample 3027), where there was much higher concentration of sENG measured in the complex than the total sENG despite the BMP9 concentration in this sample was not particularly higher. Since sENG concentration in the complex is not likely to exceed total sENG concentration within the same sample, sample 3027 was excluded from the analysis in Figure 3D. ***(C)*** Plot of sENG:BMP9 measurements without excluding sample 3027.

Figure S6. Dimeric sENG and ENG-Fc on BMP9 signaling activity. ***(A&B)*** Dimeric sENG does not inhibit BMP9 signaling in hPAECs. Serum-starved hPAECs were treated with BMP9-GFD or pro-BMP9 which has been pre-incubated with sENG(D) at indicated concentrations. Cells were harvested after 15 minutes for probing Smad1/5 phosphorylation or 1 hour for probing the changes in *ID1* and *ID2* gene expression. ***(C)*** Serum-starved hPAECs were treated with BMP9-GFD or pro-BMP9 which has been pre-incubated with sENG-Fc at indicated concentrations. Cells were harvested after 15 minutes for monitoring Smad1/5 phosphorylation. One representative blot of 5 experiments is shown, with quantifications on the right. ***(D)*** Purified sENG(D):BMP9 complex was as active as pro-BMP9. sENG(D):BMP9 was purified as shown in Figure S7B, and the amount of BMP9-GFD in the complex was quantified by SDS-PAGE and Coomassie Blue staining using pro-BMP9 as a standard. Serum-starved PAECs were treated with PBS, pro-BMP9 (0.4 ng/ml BMP9-GFD), sENG(D):BMP9 (0.4 ng/ml BMP9-GFD), or sENG(D) (67.5 ng/ml) for 1.5 hours, after which cells were harvested and *ID1* gene expression measured using RT-qPCR.

Figure S7. Flowcharts illustrating the generation of sENG(M):BMP9 complex. ***(A)*** Generation of sENG(M):BMP9 Prep 1. The goal of this experiment was to generate sENG(M):BMP9 complex that has the same concentration (or as close as possible) of BMP9-GFD as the pro-BMP9 control sample. In the sENG(M):BMP9 arm, pro-BMP9, containing 10 μg of GFD, was mixed with sENG(M) and the sENG(M):BMP9 complex was purified on a S200 gel filtration column. In the control arm, same amount of pro-BMP9 was mixed with PBS/BSA before diluted to the same volume as the sENG(M):BMP9 complex. Thus, the concentration of BMP9-GFD in the sENG:BMP9 complex will be almost identical to the pro-BMP9 control, which was later confirmed by BMP9 ELISA. ***(B)*** Generation of sENG(M):BMP9 Prep 2. The goal of this experiment was to generate sENG(M):BMP9 complex as pure as possible, and alongside sENG(M):BMP10 complex and sENG(M) control. Equal volumes of conditioned media containing sENG were loaded onto three Ni-NTA columns. For sENG:BMP9 and sENG:BMP10 arms, the two columns were subsequently loaded with purified pro-BMP9 or pro-BMP10 respectively before the washing steps. The three Ni columns were then followed the same purification steps, including washing and elution using the same imidazole gradient. Fractions were monitored by SDS-PAGE and Coomassie Blue staining, and those containing sENG were pooled and further purified on a S200 gel filtration columns. SDS-PAGE and western blots were used to monitor the fractions in the final steps of purification.

Figure S1

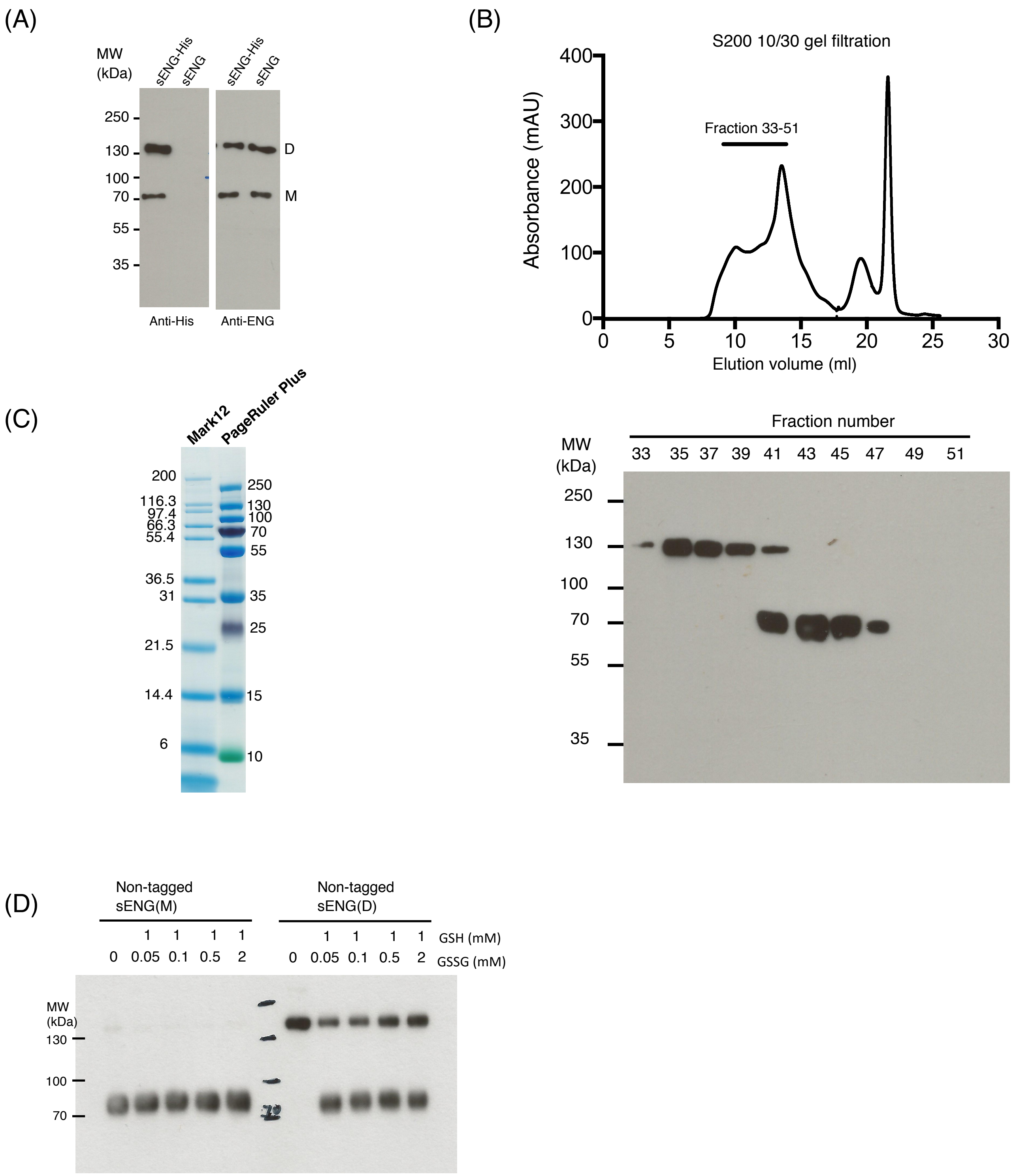

(A)
MW (kDa)
sENG-His
sENG
sENG-His
sENG
250
130
100
70
55
35
D
M
Anti-His
Anti-ENG
(B)
S200 10/30 gel filtration
Absorbance (mAU)
400
300
200
100
0
Fraction 33-51
0
5
10
15
20
25
30
Elution volume (ml)
Fraction number
MW (kDa)
33
35
37
39
41
43
45
47
49
51
250
130
100
70
55
35
(C)
Mark12
PageRuler Plus
200
116.3
97.4
66.3
55.4
36.5
31
21.5
14.4
6
250
130
100
70
55
35
25
15
10
(D)
Non-tagged sENG(M)
Non-tagged sENG(D)
1 1 1 1
1 1 1 1
GSH (mM)
0 0.05 0.1 0.5 2
0 0.05 0.1 0.5 2
GSSG (mM)
MW (kDa)
130
100
70

Figure S2

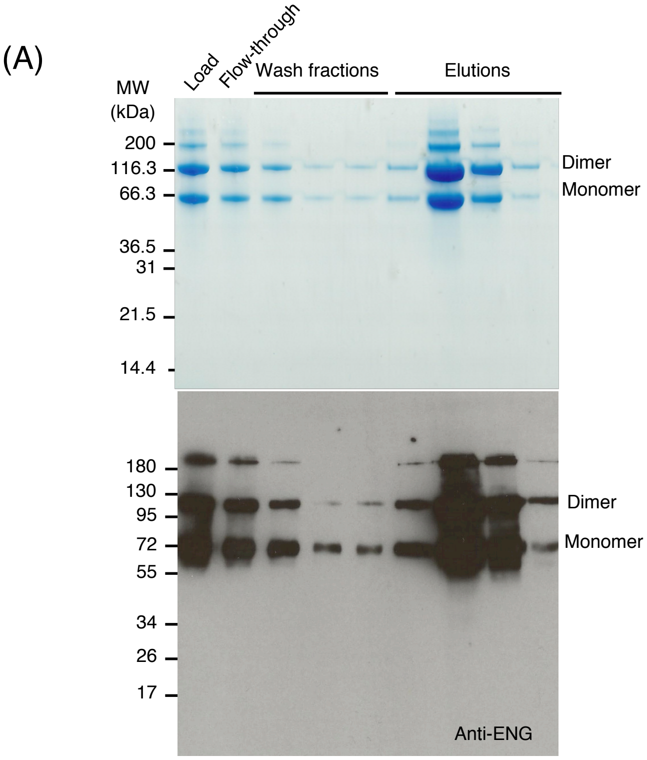
(A)
MW (kDa)
Load
Flow-through
Wash fractions
Elutions
200
116.3
66.3
36.5
31
21.5
14.4
Dimer
Monomer
180
130
95
72
55
34
26
17
Dimer
Monomer
Anti-ENG

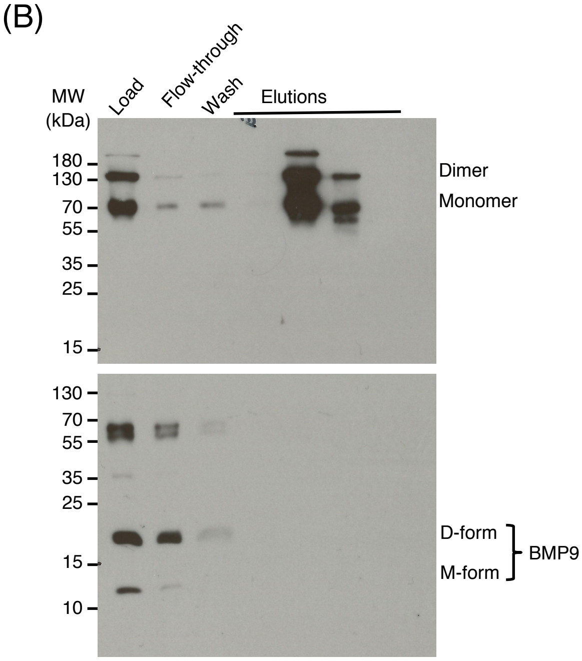
(B)
MW (kDa)
Load
Flow-through
Wash
Elutions
180
130
70
55
35
25
15
Dimer
Monomer
130
70
55
35
25
15
10
D-form
M-form
BMP9

Figure S3

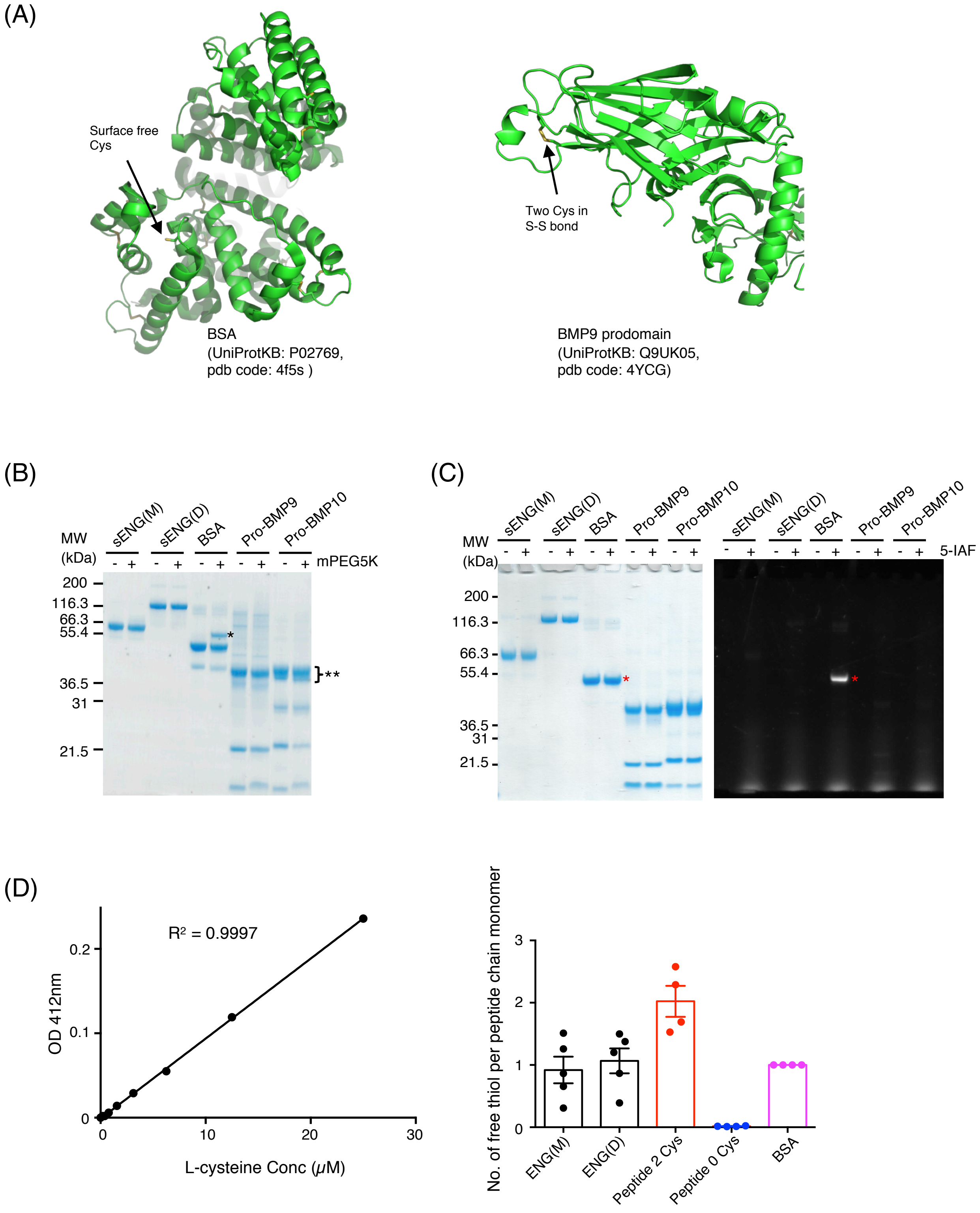
(A)
Surface free Cys
BSA
(UniProtKB: P02769,
pdb code: 4f5s)
Two Cys in S-S bond
BMP9 prodomain
(UniProtKB: Q9UK05,
pdb code: 4YCG)
(B)
MW (kDa)
sENG(M)
sENG(D)
BSA
Pro-BMP9
Pro-BMP10
- + - + - + - + - + mPEG5K
200
116.3
66.3
55.4
36.5
31
21.5
*
}**
(C)
MW (kDa)
sENG(M)
sENG(D)
BSA
Pro-BMP9
Pro-BMP10
- + - + - + - + - + - + - + - + - + - + 5-IAF
200
116.3
66.3
55.4
36.5
31
21.5
*
*
(D)
R^2 = 0.9997
OD 412nm
L-cysteine Conc (μM)
0
0.1
0.2
0
10
20
30
No. of free thiol per peptide chain monomer
0
1
2
3
ENG(M)
ENG(D)
Peptide 2 Cys
Peptide 0 Cys
BSA

Figure S4

(A)

	Test samples		
**Input protein**	sENG(M)	sENG(M):BMP9	Pro-BMP9
sENG (M)	+	+	-
Pro-BMP9	-	+	+

(B)

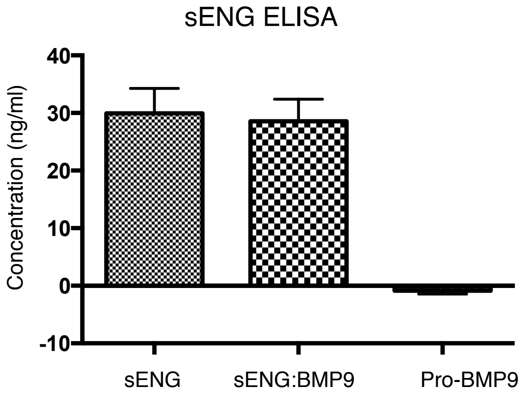

(C)

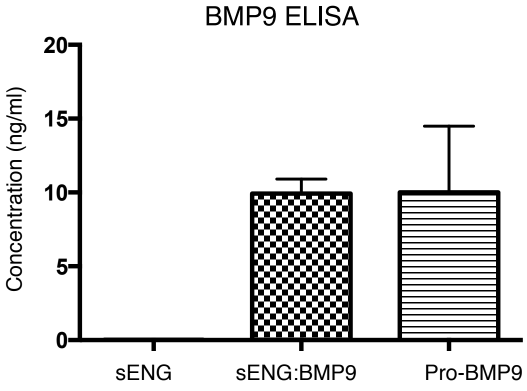

(D)

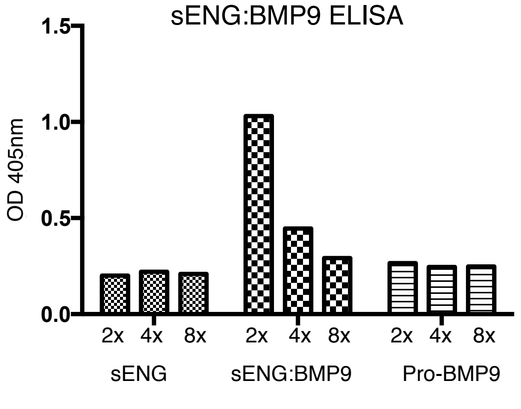

Figure S5

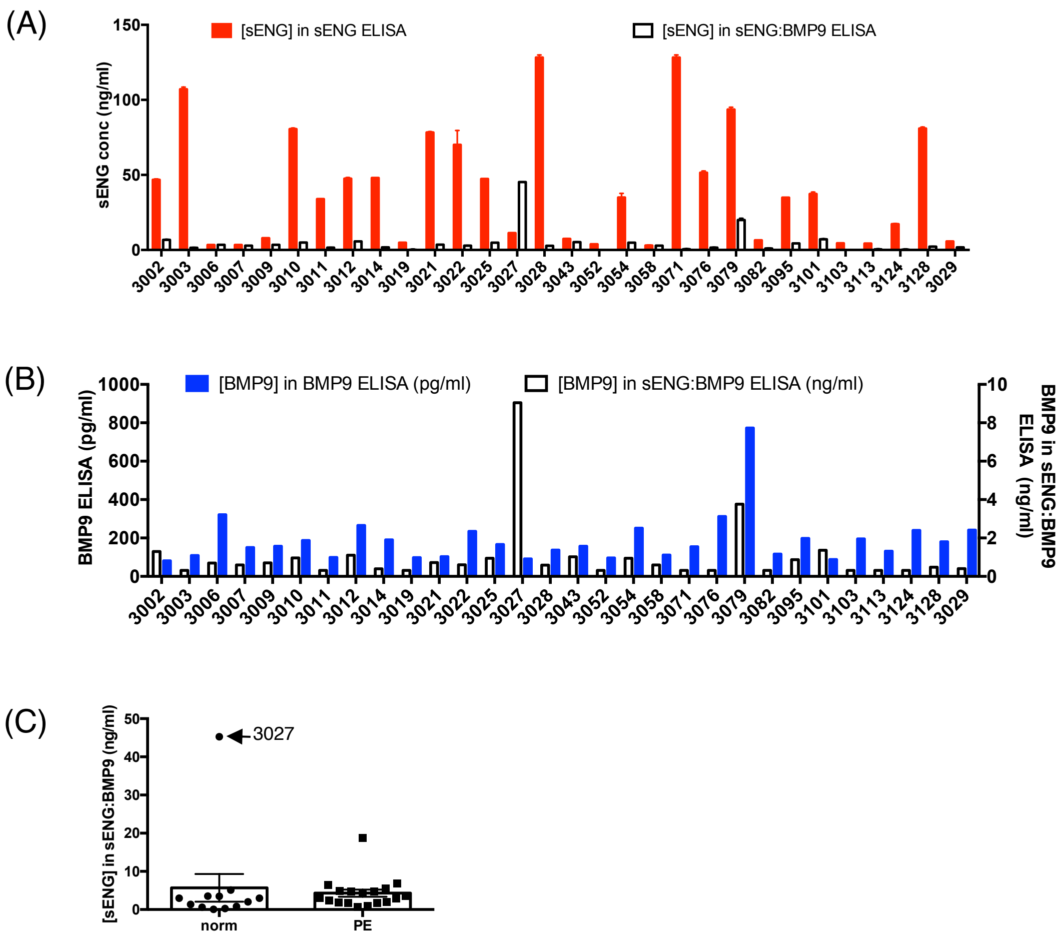

(A)
[sENG] in sENG ELISA
[sENG] in sENG:BMP9 ELISA
sENG conc (ng/ml)
150
100
50
0
3002 3003 3006 3007 3009 3010 3011 3012 3014 3019 3021 3022 3025 3027 3028 3043 3052 3054 3058 3071 3076 3079 3082 3095 3101 3103 3113 3124 3128 3029
(B)
[BMP9] in BMP9 ELISA (pg/ml)
[BMP9] in sENG:BMP9 ELISA (ng/ml)
BMP9 ELISA (pg/ml)
1000
800
600
400
200
0
BMP9 in sENG:BMP9 ELISA (ng/ml)
10
8
6
4
2
0
3002 3003 3006 3007 3009 3010 3011 3012 3014 3019 3021 3022 3025 3027 3028 3043 3052 3054 3058 3071 3076 3079 3082 3095 3101 3103 3113 3124 3128 3029
(C)
[sENG] in sENG:BMP9 (ng/ml)
50
40
30
20
10
0
3027
norm
PE

Figure S6

(A)

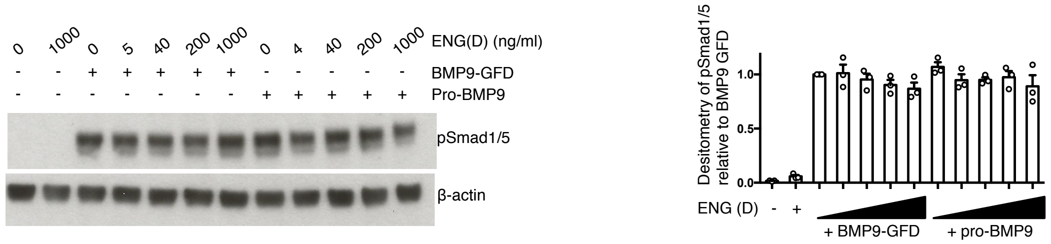

(B)

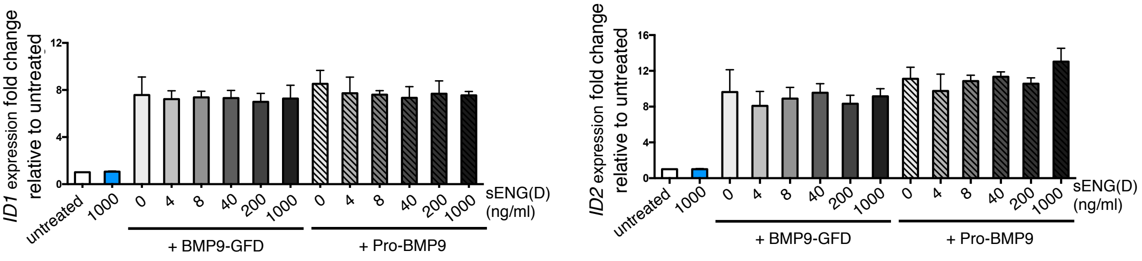

(C)

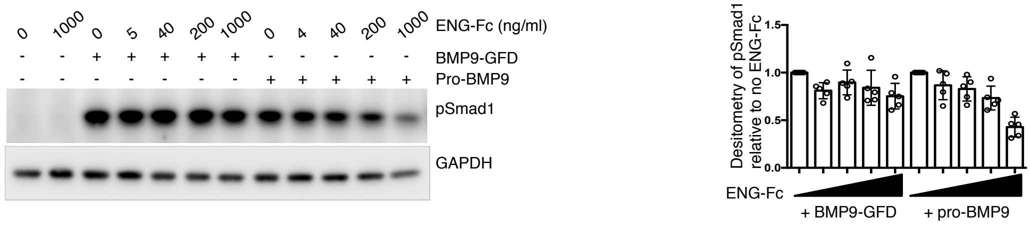

(D)

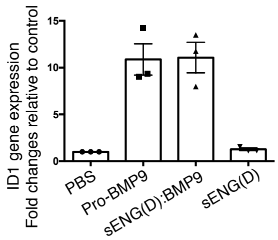

Figure S7

(A) sENG(M):BMP9 prep1

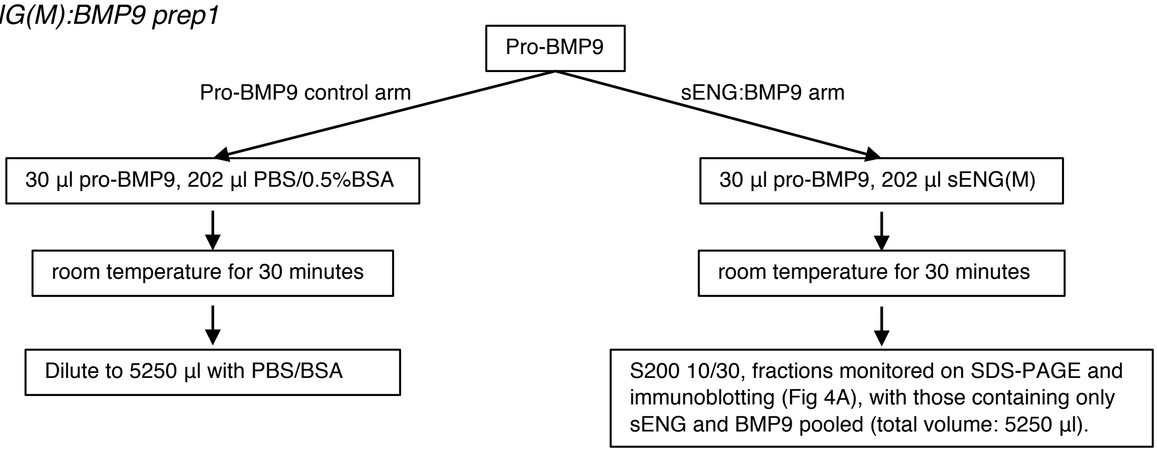

(B) sENG(M):BMP9 prep2

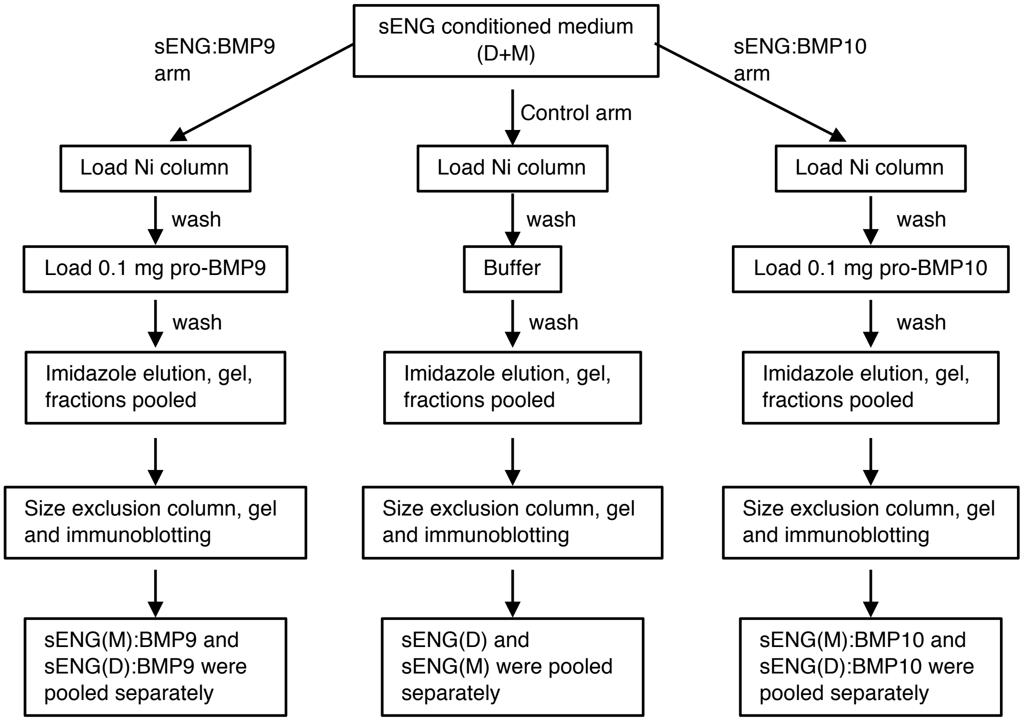

Supplement: Supplementary File [file pnas.1816661116.sapp.pdf]
